# Interleukin-1 signaling induced by *Streptococcus suis* serotype 2 is strain-dependent and contributes to bacterial clearance and inflammation during systemic disease in a mouse model of infection

**DOI:** 10.1186/s13567-019-0670-y

**Published:** 2019-07-01

**Authors:** Agustina Lavagna, Jean-Philippe Auger, Audrey Dumesnil, David Roy, Stephen E. Girardin, Nicolas Gisch, Mariela Segura, Marcelo Gottschalk

**Affiliations:** 1 Research Group on Infectious Diseases in Production Animals (GREMIP) and Swine and Poultry Infectious Diseases Research Center (CRIPA), Department of Pathology and Microbiology, Faculty of Veterinary Medicine, University of Montreal, Saint-Hyacinthe, QC Canada; 2 Department of Laboratory Medicine and Pathobiology, University of Toronto, Toronto, ON Canada; 3 Division of Bioanalytical Chemistry, Priority Area Infections, Research Center Borstel, Leibniz Lung Center, Borstel, Germany

## Abstract

*Streptococcus suis* serotype 2 is an important porcine pathogen and zoonotic agent causing sudden death, septic shock and meningitis, with exacerbated inflammation being a hallmark of the infection. A rapid, effective and balanced innate immune response against *S. suis* is critical to control bacterial growth without causing excessive inflammation. Even though interleukin (IL)-1 is one of the most potent and earliest pro-inflammatory mediators produced, its role in the *S. suis* pathogenesis has not been studied. We demonstrated that a classical virulent European sequence type (ST) 1 strain and the highly virulent ST7 strain induce important levels of IL-1 in systemic organs. Moreover, bone marrow-derived dendritic cells and macrophages contribute to its production, with the ST7 strain inducing higher levels. To better understand the underlying mechanisms involved, different cellular pathways were studied. Independently of the strain, IL-1β production required MyD88 and involved recognition via TLR2 and possibly TLR7 and TLR9. This suggests that the recognized bacterial components are similar and conserved between strains. However, very high levels of the pore-forming toxin suilysin, produced only by the ST7 strain, are required for efficient maturation of pro-IL-1β via activation of different inflammasomes resulting from pore formation and ion efflux. Using IL-1R^−/−^ mice, we demonstrated that IL-1 signaling plays a beneficial role during *S. suis* systemic infection by modulating the inflammation required to control and clear bacterial burden, thus promoting host survival. Beyond a certain threshold, however, *S. suis*-induced inflammation cannot be counterbalanced by this signaling, making it difficult to discriminate its role.

**Electronic supplementary material:**

The online version of this article (10.1186/s13567-019-0670-y) contains supplementary material, which is available to authorized users.

## Introduction

*Streptococcus suis* causes sudden death and meningitis in pigs and is responsible for important economic losses to the swine industry. Furthermore, it is also a zoonotic agent responsible for meningitis and septic shock in humans [1, 2]. Of the 35 described serotypes, serotype 2 is the most virulent and most frequently isolated from both pigs and humans worldwide [3]. Using multilocus sequence typing, four predominant sequence types (STs) have been identified within serotype 2 strains: the virulent ST1 in Eurasia, the highly virulent ST7 responsible for two human outbreaks in China and the intermediate and low virulent ST25 and ST28, respectively, in North America [4]. A variety of virulence factors have been proposed to be involved in the *S. suis* pathogenesis, including capsular polysaccharide, lipoproteins (LPs) and lipoteichoic acid (LTA) modifications [5]. Moreover, ST1 and ST7 strains produce suilsyin (SLY), a hemolysin similar to the pneumolysin of *Streptococcus pneumoniae* and which participates in bacterial dissemination and host inflammation [6, 7].

A rapid and effective innate immune response against *S. suis* is critical to control bacterial growth and limit the spread of the pathogen [8]. Recognition by specialized membrane-associated or cytoplasmic receptors (pattern recognition receptors [PRRs]) mediates host immune responses by inducing mediator production via activation of nuclear factor-kappa B (NF-ĸB) and mitogen-activated protein kinases (MAPKs) [9]. Previous studies have shown that *S. suis* activates dendritic cells (DCs) and macrophages (MФ) through the myeloid differentiation primary response 88 (MyD88)-dependent Toll-like receptor (TLR) pathway [10–12]. Indeed, recognition of *S. suis* occurs via surface-associated TLR2 and, possibly, TLR4 [13], while its internalization activates the endosomal TLR7 and TLR9 [14].

Of the different mediators induced during inflammation, interleukin (IL)-1 is one of the most potent and earliest produced, of which there exist two forms (IL-1α and IL-1β) encoded by separate genes and synthesized as precursor peptides (pro-IL-1α and pro-IL-1β) [15]. While pro-IL-1α is biologically active, a two steps mechanism is required for the complete maturation of IL-1β [16, 17]. Firstly, activation of PRRs leads to transcription and translation of pro-IL-1β, which is then cleaved and activated by caspase-1-dependent mechanisms [18]. Moreover, caspase-1 itself requires proteolytic processing mediated by intracellular complexes called inflammasomes. Though several inflammasomes have been described, the nucleotide-binding oligomerization domain (NOD)-like receptor (NLR) family pyrin domain-containing 3 (NLRP3), the NLRP1, the NLR family CARD domain-containing protein 4 (NLRC4), and the absent in melanoma 2 (AIM2) are the best characterized [19, 20]. Once secreted, IL-1α and IL-1β bind to their shared receptor, IL-1 receptor (IL-1R), which is ubiquitously expressed, resulting in the recruitment of inflammatory cells and their activation [17].

Although IL-1 signaling plays an essential role in in the initiation of the inflammatory response, an uncontrolled production of IL-1 can lead to tissue damage and disease. While, IL-1 plays a protective role during both pneumococcal and Group B *Streptococcus* (GBS) infections [21–24], its role is detrimental in a mouse model of Group A *Streptococcus* (GAS) infection [25, 26]. During *S. suis* infection, the host response depends on the ability of innate immune mechanisms to control initial bacterial growth and limit its spread without causing excessive inflammation. However, regardless of the numerous studies on the *S.* *suis* pathogenesis, none have focused on the production and role of IL-1. Consequently, we assessed herein its implication during *S. suis* serotype 2 pathogenesis using a classical virulent European ST1 strain and the highly virulent ST7 strain by evaluating the mechanisms involved in its production in vitro and its role in vivo during the systemic infection.

## Materials and methods

### Bacterial strains, plasmids and growth conditions

The strains and plasmids used in this study are listed in Table 1. The classical virulent European reference ST1 P1/7 strain and the highly virulent Chinese ST7 SC84 strain were used throughout this study, including for construction of isogenic *sly*-deficient mutants. *S. suis* strains were grown in Todd Hewitt broth (THB; Becton–Dickinson, Mississauga, ON, Canada) [27], diluted in culture medium before experiments with cells, and the final concentration (colony-forming units [CFU]/mL) determined by plating on THB agar (THA). For experimental mouse infections, bacteria were resuspended in THB. *Escherichia* *coli* was grown in Luria–Bertani (LB) broth or agar (Becton–Dickinson). When needed, antibiotics (Sigma-Aldrich, Oakville, ON, Canada) were added to culture media at the following concentrations: for *E. coli*, ampicillin at 100 mg/mL, kanamycin and spectinomycin at 50 μg/mL; for *S. suis*, spectinomycin at 100 μg/mL.

**Table 1:** **Bacterial strains and plasmids used in this study**

Strain/plasmid	General characteristics	Source/references
*Streptococcus suis*		
P1/7	Classical virulent serotype 2 ST1 strain isolated from a pig with meningitis in the UK	[69]
P1/7Δ*sly*	Isogenic mutant strain derived from P1/7; in frame deletion of *sly* gene	This work
P1/7Δ*lgt*	Isogenic mutant strain derived from P1/7; in frame deletion of *lgt* gene	[31]
SC84	Highly virulent serotype 2 ST7 strain isolated from a human case of streptococcal toxic shock-like syndrome during the 2005 human outbreak in China	[70]
SC84Δ*sly*	Isogenic mutant strain derived from SC84; in frame deletion of *sly* gene	This work
SC84Δ*lgt*	Isogenic mutant strain derived from SC84; in frame deletion of *lgt* gene	[31]
*Escherichia coli*		
TOP 10	F^−^ mrcA Δ(mrr-hsdRMS-mcrBC) φ80 lacZΔM15 ΔlacX74 recA1 araD139 Δ(ara-leu) 7697 galU galK rpsL (Str^R^) endA1 nupG	Invitrogen
BL21	F^−^ompT hsdS_B_ (r_B_^−^, m_B_^−^) gal dcm rne131 (DE3)	Invitrogen
Plasmids		
pIVEX2.4d	Ap^r^, pUC ori, T7 promoter, His tag-coding sequence	Roche Bioscience
pSET4Δ*sly*	pSET4 s carrying the construct of *sly* gene for allelic replacement	This work
pIVEX*sly*	pET101 carrying *sly* gene for protein production	This work

### Construction of the isogenic *sly*-deficient mutants

Precise in-frame deletion of the *sly* gene from *S. suis* strains P1/7 and SC84 was achieved using splicing-by-overlap-extension polymerase chain reaction (PCR) [28]. Oligonucleotide primers (Additional file 1) were obtained from Integrated DNA Technologies (Coralville, IA, USA) and PCRs were carried out with the iProof proofreading DNA polymerase (BioRad Laboratories, Mississauga, ON, Canada) or with the Taq DNA polymerase (Qiagen, Valencia, CA, USA). Overlapping PCR-products were cloned into the plasmid pCR2.1 (Invitrogen, Burlington, ON, Canada), extracted using EcoRI, and cloned into the thermosensitive *E. coli*–*S. suis* shuttle vector pSET4s [29]. Final constructions of pSET4s vectors were electroporated into competent *S. suis* as previously described [29]. Deletion of the *sly* gene was confirmed by PCR and sequencing.

### Cloning, expression and purification of recombinant suilysin (rSLY)

The *sly* gene from *S. suis* strain P1/7, excluding the signal peptide, was amplified by PCR, after which amplicons were digested with *Nde*I and *Bam*HI and cloned into the pIVEX2.4d vector (Roche, Mississauga, ON, Canada), which possesses an N-terminal His-tag. Primers used are listed in Additional file 1. Protein synthesis was induced in the *E. coli* BL21 (DE3) strain using 0.5 mM isopropyl-β-d-thiogalactopyranoside for 4 h, after which cells were sonicated. The resulting recombinant His-tag suilysin (rSLY) was purified by affinity chromatography using the HisPur Ni-NTA Spin Column Kit (Thermo Scientific, Rochelle, IL, USA) according to manufacturer’s instructions. rSLY kept its hemolytic activity as determined using red blood cells (see below). Protein quantification was measured using the Pierce Bicinchoninic Acid Protein Assay Kit (Thermo Scientific).

### Titration of hemolytic activity

Hemolytic titration was performed by preparing twofold serial dilutions of bacterial culture supernatant in a solution of 0.145 M NaCl and 7 mM Na_2_HPO_4_, pH 7.2, using horse red blood cells as previously described [30]. The titer was defined as the reciprocal of the highest dilution with observed hemolysis.

### Lipoteichoic acid preparation

Extraction and purification of LTA from strains P1/7, P1/7Δ*lgt*, SC84, and SC84Δ*lgt* has recently been described [31].

### Mice

MyD88^−/−^ [B6.129P2(SJL)-MyD88^*tm1.Defr*^/J], TRIF^−/−^ [C57BL/6 J-Ticam1^*Lps2*^/J], TLR2^−/−^ [B6.129-Tlr2^*tmKir*^/J], TLR4^−/−^ [B6.B10ScN-Tlr4^*lps*−*del*^/JthJ], caspase-1^−/−^ [B6N.129S2-*Casp1*^*tm1Flv*^/J], NLRP3^−/−^ [B6.129S6-*Nlrp3*^*tm1Bhk*^/J], NLRP1^−/−^ [B6.129S6-*Nlrp1b*^*tm1Bhk*^/J], AIM2^−/−^ [B6.129P2-*Aim2*^*Gt(CSG445)Byg*^/J], NLRC4^−/−^ [32], and IL-1R^−/−^ [B6.129S7-*Il1r1*^*tm1Imx*^/J] mice on C57BL/6 background were housed under specific pathogen-free conditions alongside their wild-type counterparts. Mice were purchased from Jackson Research Laboratories (Bar Harbor, ME, USA), except for NLRC4^−/−^ mice, generated by Dr. G. Núñez (University of Michigan, USA) [33].

### Generation of bone marrow-derived dendritic cells and macrophages

Hematopoietic stem cells from femurs and tibias of wild-type and knockout mice were used to generate bone marrow-derived DCs (bmDCs) as previously described [11, 14] in RPMI-1640 supplemented with 5% heat-inactivated fetal bovine serum, 10 mM HEPES, 2 mM l-glutamine, and 50 µM 2-mercaptoethanol (all from Gibco, Burlington, ON, Canada) and complemented with 10% granulocyte–macrophage colony-stimulating factor. For macrophages (MΦ), hematopoietic stem cells (5 × 10^5^ cells/mL) were cultured in Dulbecco’s Modified Eagle’s Medium supplemented with 10% heat-inactivated fetal bovine serum (Gibco) and complemented with 30% L929 cell-derived macrophage colony-stimulating factor supernatant [34]. Cells were cultured for 8 days at 37 °C with 5% CO_2_ and trypsinized using 0.05% trypsin-0.03% EDTA (Gibco) prior to infection. Cell purity was at least 85% CD11c^+^ or F4/80^+^ for bmDCs and MΦ, respectively. Albeit this culture system cannot completely rule out the presence of other innate cells (such as macrophages), it represents an enriched source of bmDCs.

### *S. suis* infection of bone marrow-derived dendritic cells and macrophages

All activation studies were done with endotoxin-free material and under non-toxic conditions as evaluated using the CytoTox 96^®^ Non-Radioactive Cytotoxicity Assay (Promega, Madison, WI, USA). Cells were resuspended at 1 × 10^6^ cells/mL in complete medium and stimulated with the different *S. suis* serotype 2 strains listed in Table 1 (1 × 10^6^ CFU/mL; initial multiplicity of infection = 1). Conditions used were based on those previously published [6, 11]. At indicated times, supernatants were collected for cytokine measurement. For mRNA expression, cells were harvested in TRIzol (Invitrogen) 6 h following infection. Mock-infected cells served as negative controls. Activation of cells with LTA was performed using 30 μg/mL and supernatants collected 24 h later for IL-1β quantification. For signaling pathway studies, cells were pretreated for 30 min with 10 μM NF-κB inhibitor JSH-23, 10 μM p38 inhibitor SB0203580, 25 μM MEK1/2 inhibitor U0126 or 10 μM JNK inhibitor SP600125 (all from Calbiochem/EMD Millipore, San Diego, CA, USA), in dimethyl sulfoxide (DMSO; Sigma-Aldrich), which served as the vehicle. For assays with rSLY, a non-toxic concentration of 5 μg/mL was used during 16 h. For cholesterol inhibition assays, 40 μg/mL of cholesterol (Sigma-Aldrich) was added to wells [30]. When needed, 0.25 mg/mL alhydrogel (Brenntag, Mülheim, Germany) was added as a NLRP3 activator immediately upon infection of cells. Finally, for experiments involving extracellular K^+^, a stock solution of KCl (Laboratoire Mat. Inc., Quebec City, QC, Canada) was prepared and appropriately diluted.

### *S. suis* DNA and RNA preparation and transfection of cells

Bacterial DNA and RNA isolation was performed as previously described [14]. Cells were transfected with 1 μg of RNA or DNA complexed or not with DOTAP liposomal transfection reagent (Sigma-Aldrich) as previously described [14].

### Cytokine and chemokine quantification in cell culture supernatants

Levels of IL-1α, IL-1β, IL-6, and tumor necrosis factor (TNF) in cell culture supernatants were measured by sandwich enzyme-linked immunosorbent assay (ELISA) using pair-matched antibodies from R&D Systems (Minneapolis, MN, USA) according to the manufacturer’s recommendations.

### Determination of cell mRNA expression by RT-qPCR

Cell mRNA was extracted according to the manufacturer’s instructions (TRIzol). RNA purity was assessed by spectrophotometric quantification and integrity verified by electrophoresis on denaturating agarose gel. cDNA was generated using the Quantitect cDNA Synthesis Kit (Qiagen, Mississauga, ON, Canada) with 500 ng of RNA pretreated with DNase. Real-time qPCR was performed on the CFX-96 Touch Rapid Thermal Cycler System (Bio-Rad) using 250 nM of primers (Integrated DNA technologies), the SsoFast Evagreen Supermix Kit (Bio-Rad) and 20 ng of cDNA. No template controls were included and all samples were run in triplicate. The cycling conditions were 3 min of polymerase activation at 98 °C, followed by 40 cycles at 98 °C for 2 s and 57 °C for 5 s. Melting curves were generated after each run to confirm the presence of a single PCR product. The sequences of primers used in this study are shown in Additional file 1 and were verified to have reaction efficiencies between 90 and 110%. The reference genes *Atp5b* and *Gapdh*, determined to be the most stably expressed using the algorithm geNorm, were used to normalize data. Fold changes in gene expression were calculated using the quantification cycle threshold (Cq) method using the CFX software manager v.3.0 (Bio-Rad). Samples from mock-infected cells served as calibrators.

### *S. suis* serotype 2 mouse model of infection

Six-week-old male and female wild-type C57BL/6 and IL-1R^−/−^ mice were used. Animals were acclimatized to standard laboratory conditions with unlimited access to water and rodent chow [35]. These studies were carried out in strict accordance with the recommendations of and approved by the University of Montreal Animal Welfare Committee guidelines and policies, including euthanasia to minimize animal suffering, applied throughout this study when animals were seriously affected since mortality was not an end-point measurement. The different *S. suis* serotype 2 strains or the vehicle solution (sterile THB) were administered at a dose of 1 × 10^7^ CFU by intraperitoneal inoculation. Survival was evaluated and mice monitored at least three times daily until 7 days post-infection (pi).

### Measurement of plasma, liver and spleen pro-inflammatory mediators

For kinetics of IL-1α and IL-1β production, blood from infected mice was collected by intracardiac puncture following euthanasia and anti-coagulated with EDTA (Sigma-Aldrich) as previously described [8, 36]. Plasma was collected following centrifugation at 10 000 × *g* for 10 min at 4 °C. For liver and spleen, extraction buffer was prepared using complete Mini, EDTA-free, protease inhibitor cocktail tablets (Roche Diagnostics GmbH, Mannheim, Germany) according to the manufacturer’s instructions and organs homogenized using a POLYTRON PT 1200E system bundle (Kinematica, Lucerne, Switzerland). Homogenate supernatants were collected following centrifugation at 10 000 × *g* for 10 min at 4 °C. Levels of IL-1α and IL-1β were determined by ELISA, while IL-6, IL-12p70, interferon (IFN)-γ, C–C motif chemokine ligand (CCL) 2, CCL3, and C–X–C motif chemokine ligand (CXCL) 9 were measured using a custom-made cytokine Bio-Plex Pro™ assay (Bio-Rad). Acquisition was performed on the MAGPIX platform (Luminex^®^) and data analyzed using the Bio-Plex Manager 6.1 software (Bio-Rad).

### Measurement of blood, spleen and liver bacterial burden

Wild-type and IL-1R^−/−^ mice were inoculated with *S. suis* as described above and blood bacterial burden was assessed 12 h and 48 h pi by collecting blood from the caudal vein. For liver and spleen, organs were collected and homogenized as described above. Bacterial burden was determined by plating appropriate dilutions on THA.

### Statistical analyses

Normality of data was verified using the Shapiro–Wilk test. Accordingly, parametric (unpaired t-test) or non-parametric tests (Mann–Whitney rank sum test), where appropriate, were performed to evaluate statistical differences between groups. Log-rank (Mantel–Cox) tests were used to compare survival between wild-type and IL-1R^−/−^ mice. Each test was repeated in at least three independent experiments. *p *< 0.05 was considered as statistically significant.

## Results

### *S. suis* serotype 2 induces elevated levels of IL-1 in organs but not in plasma

**Figure 1:**
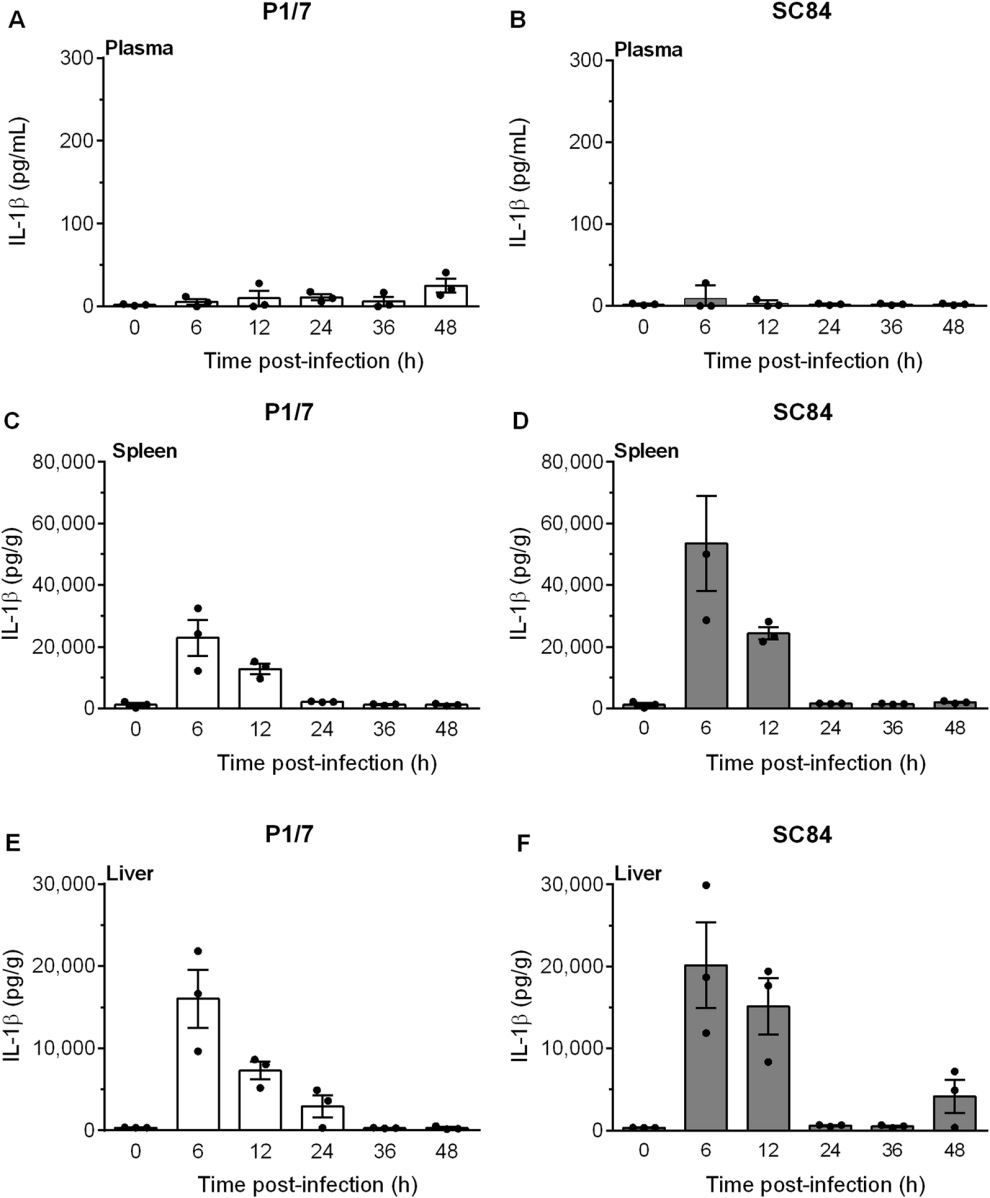
***S. suis*** **induces elevated levels of IL-1β in liver and spleen but not in plasma.** C57BL/6 mice were intraperitoneally inoculated with the *S. suis* strain P1/7 (white bars) or SC84 (gray bars). Plasma (**A**, **B**), spleen (**C**, **D**) and liver (**E**, **F**) were collected at different times post-infection and levels of IL-1β were quantified by ELISA. Values for mock-infected controls did not change between 6 and 48 h. As such, 0 h represents results for mock-infected mice throughout the experiment. Data are expressed as mean ± SEM (*n* = 3).

**Figure 2:**
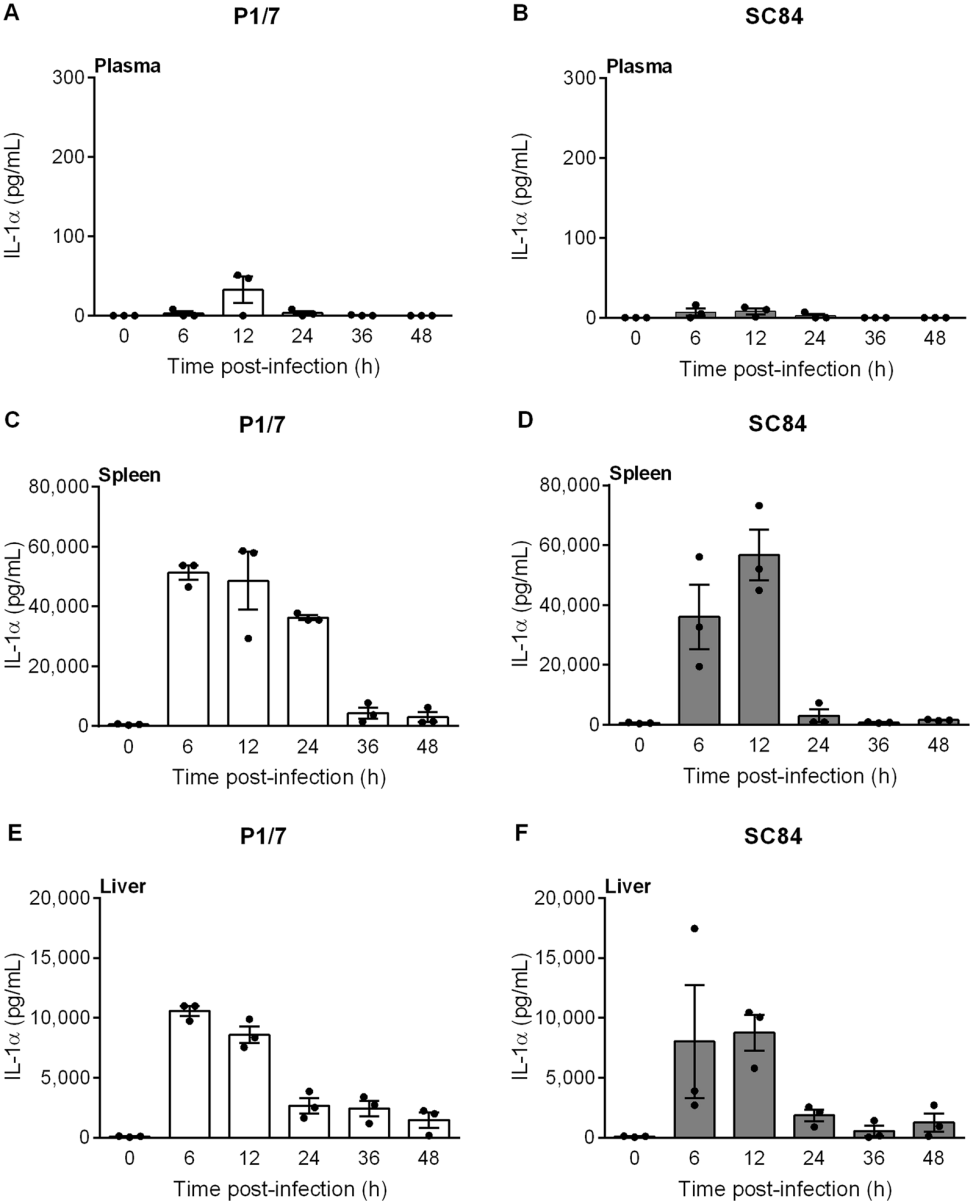
***S. suis*** **serotype 2 induces elevated levels of IL-1α in liver and spleen, but not in plasma.** C57BL/6 mice were intraperitoneally inoculated with *S. suis* strain P1/7 (white bars) or SC84 (gray bars). Plasma (**A**, **B**), spleen (**C**, **D**), liver (**E**, **F**) were collected at different times post-infection and levels of IL-1α were quantified by ELISA. Values for mock-infected controls did not change between 6 and 48 h. As such, 0 h represents results for mock-infected mice throughout the experiment. Data are expressed as mean ± SEM (*n* = 3).

Systemic levels of both IL-1α and IL-1β were measured in plasma, liver and spleen following infection with the classical European ST1 strain P1/7 and the highly virulent ST7 strain SC84. IL-1β levels were barely detectable in mock-infected animals and did not change between 6 to 48 h. Similarly, IL-1β levels were scarcely detectable in plasma throughout the course of infection, including upon presentation of severe clinical signs of systemic disease, with no significant differences between strains (Figures 1A and B). By contrast, levels of IL-1β in liver and spleen were very high and reached maximum values during the first 12 h pi, rapidly decreasing thereafter, with similar production patterns in both organs and between strains (Figures 1C–F). Moreover, IL-1α production patterns in plasma, liver and spleen were similar to IL-1β (Figure 2).

### *S. suis* induces IL-1 release from bone marrow-derived dendritic cells and macrophages in a strain-dependent manner

**Figure 3:**
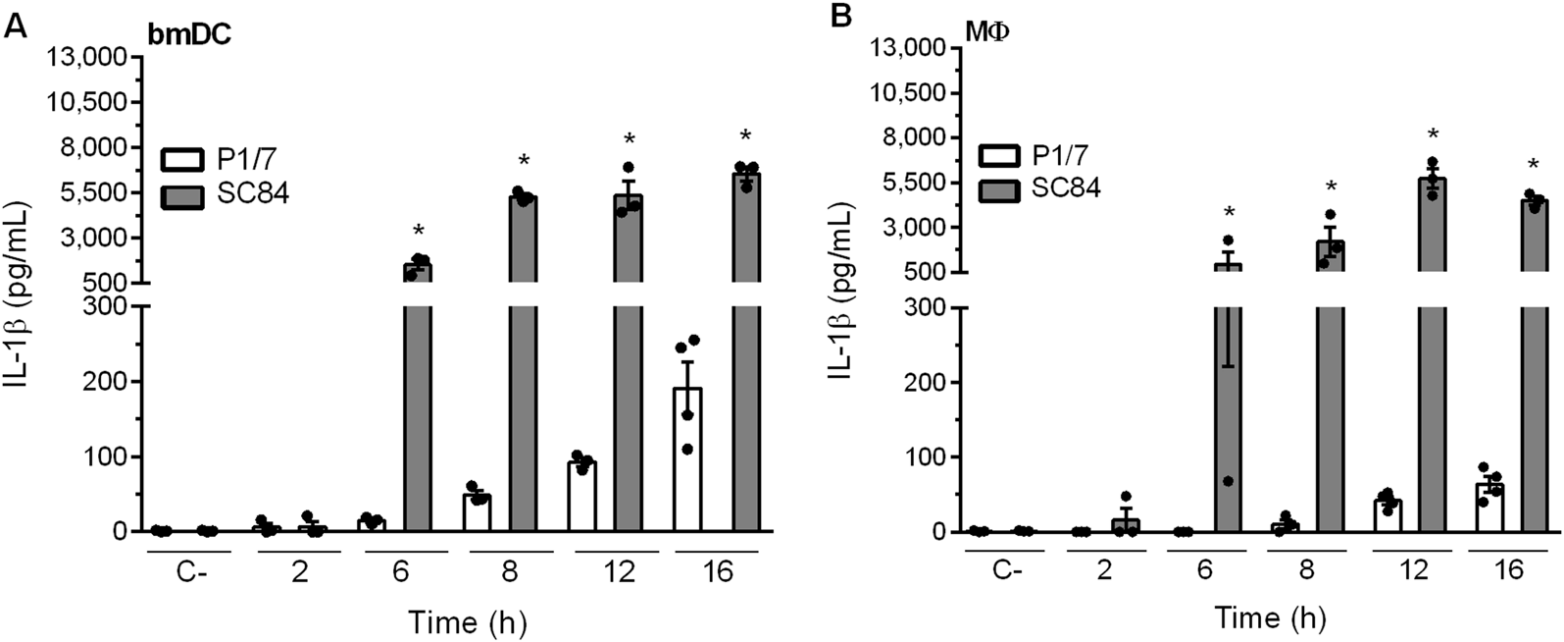
**IL-1β released from bone marrow-derived dendritic cells (bmDCs) and macrophages (MФ) stimulated with** ***S. suis*** **is strain-dependent.** IL-1β kinetics as measured by ELISA following infection of bmDCs (**A**) or MФ (**B**) with strain P1/7 (white bars) or SC84 (gray bars). Non-stimulated cells served as negative control (C-). Data are expressed as mean ± SEM (*n* = 4).

Given the elevated levels of IL-1 produced in liver and spleen and the fact that DCs and MФ are highly important during *S. suis* infection [11, 14], the capacity of these cells to produce IL-1 following *S. suis* infection was evaluated. Strain P1/7 induced modest levels of IL-1β in a time-dependent manner, with production by bmDCs being significantly greater than that by MФ at 12 h and 16 h (*p *< 0.001) (Figure 3). On the other hand, the highly virulent ST7 strain SC84 induced significantly higher levels of IL-1β compared to strain P1/7 (Figure 3) at 8 h, 12 h and 16 h pi (*p* < 0.05) in both cell types. In fact, levels induced by strain SC84 16 h pi were approximately 40 times higher than those induced by strain P1/7. Interestingly, a slight delay in IL-1β production was observed by SC84-stimulated MФ in comparison to bmDCs. Moreover, IL-1α kinetics by bmDCs and MФ were similar to those obtained for IL-1β (Additional file 2). Importantly, production was not the result of cell death, since toxicity levels remained low (data not shown). Given that IL-1α and IL-1β production kinetics were similar and that both cell types responded similarly, subsequent experiments were only performed for IL-1β using bmDCs following 16 h of infection.

### Role of Toll-like receptors and associated signaling pathways in *S. suis*-induced IL-1β production

**Figure 4:**
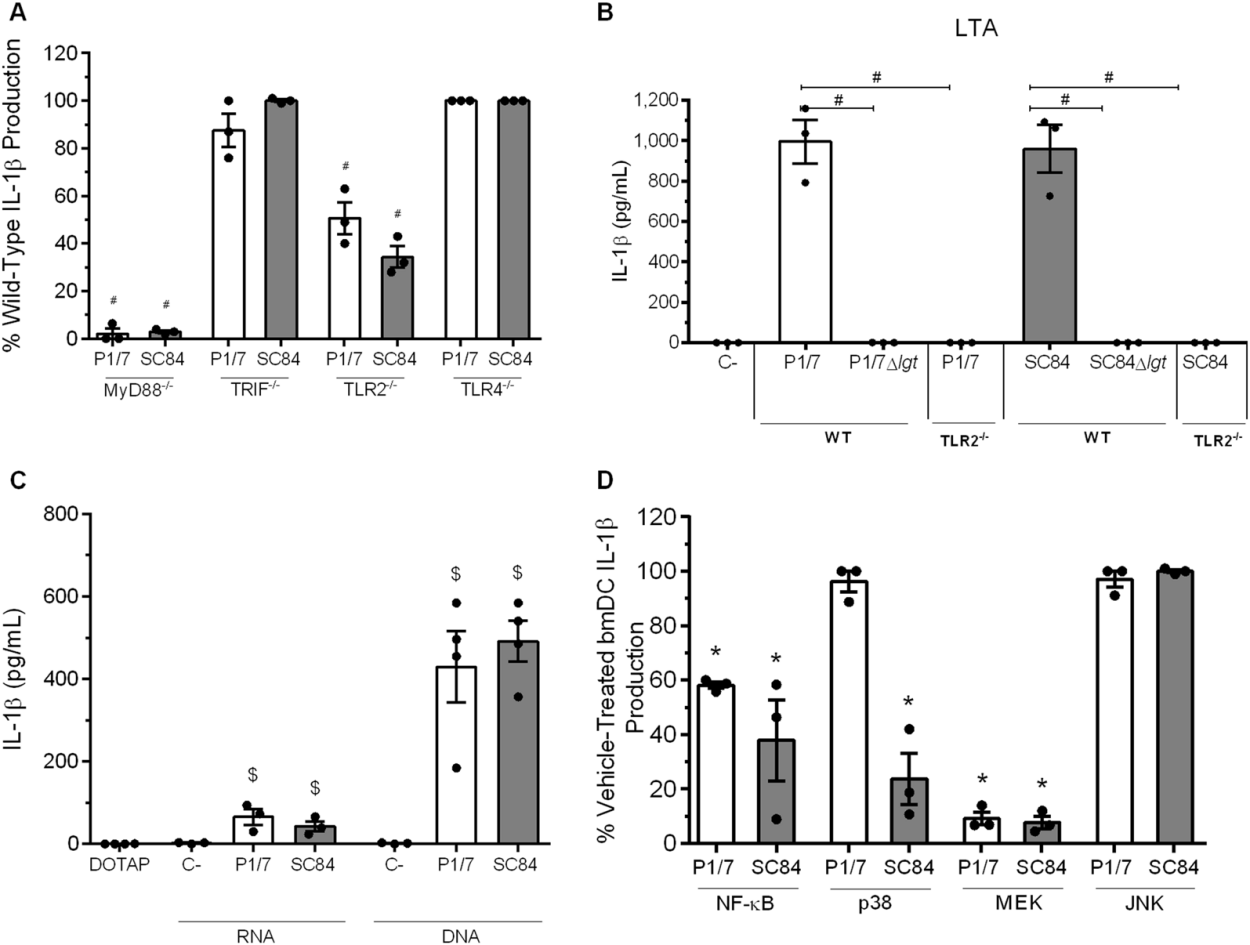
**Role of Toll-like receptors (TLRs) and associated signaling in** ***S. suis*-induced IL-1β production by bone marrow-derived dendritic cells (bmDCs)**. **A** Percentage of IL-1β production induced by *S. suis* strain P1/7 (white bars) or SC84 (gray bars) 16 h following infection of bmDCs deficient for MyD88, TRIF, TLR2 or TLR4, with regards to wild-type counterparts (normalized to 100%); **B** IL-1β production 24 h following activation of wild-type (WT) or TLR2^−/−^ bmDCs with 30 μg/mL of LTA extracts from strains P1/7 or SC84 or their *lgt*-deficient mutants (Δ*lgt*); **C** IL-1β production by bmDCs following phagosomal delivery of 1 µg of *S. suis* RNA or DNA; **D** Percentage of IL-1β production from bmDCs following pre-treatment with inhibitors of either NF-kB, p38, MEK or JNK and infection with *S. suis*, with regards to vehicle-treated bmDCs (DMSO; normalized to 100%). Data are expressed as mean ± SEM (*n* = 3). ^#^*p* < 0.05) indicates a significant difference with wild-type bmDCs (normalized to 100%); ^$^(*p* < 0.05) indicates a significant difference with negative control (elution buffer); *(*p* < 0.05) indicates a significant difference with vehicle-treated bmDCs (normalized to 100%).

To better understand the differential IL-1β production induced by strains P1/7 and SC84, the role of different receptors and signaling pathways involved in this production was evaluated. Production of IL-1β was almost completely abrogated in the absence of MyD88 following infection with both *S. suis* strains (*p* < 0.01) (Figure 4A). By contrast, production was independent of the TIR-domain-containing adapter-inducing IFN-β (TRIF) (Figure 4A). Since *S. suis* is an extracellular pathogen, its recognition by surface-associated receptors is crucial. While IL-1β production was significantly (but not totally) reduced in TLR2^−/−^ bmDCs stimulated with P1/7 or SC84 (*p* < 0.01), no difference was observed with TLR4^−/−^ bmDCs (Figure 4A).

Subsequently, potential activators of TLR2 were investigated: though still controversial, the LTA and LPs of Gram-positive bacteria have been suggested to be activators of TLR2 [37, 38]. Consequently, LTA was extracted from both strains and used to activate bmDCs, with LTA from strains P1/7 and SC84 inducing similarly high levels of IL-1β (Figure 4B). However, current methods are unable to eliminate co-purified LPs from LTA preparations [39]. As such, LTA was also extracted from *lgt*-deficient mutants (Δ*lgt*), in which absence of the lipoprotein diacylglyceryl transferase, a key enzyme in LP synthesis, renders LPs unrecognizable by TLR2 [40]. In accordance, not only did LTA preparations from *lgt*-deficient mutants induce significantly less IL-1β than those from wild-type strains (*p *< 0.01), but levels were in fact undetectable (Figure 4B). In addition, IL-1β production was completely abolished in TLR2^−/−^ bmDCs following activation with LTA preparations from wild-type strains (*p* < 0.01) (Figure 4B), indicating that co-purified LPs, but not *S.* *suis* LTA, are important inducers of IL-1β by bmDCs via recognition by TLR2.

Though an extracellular bacterium, dependence of *S. suis*-induced IL-1β production on MyD88, but only partially on TLR2 (and not at all on TLR4), suggested a potential role of endosomal TLRs. To evaluate this, DNA and RNA were extracted from strains P1/7 and SC84 and complexed or not with DOTAP liposomal transfection reagent, which allows phagosomal delivery. In the absence of DOTAP *S.* *suis* DNA and RNA did not induce IL-1β production from bmDCs (data not shown). Meanwhile, DNA and RNA from both strains induced significant, but similar production when complexed with DOTAP (*p *< 0.05) (Figure 4C). At the same concentration, *S.* *suis* DNA induced greater IL-1β production than RNA (*p* < 0.01). When Alum was added, a known activator of the NLRP3 inflammasome, a significantly higher production was observed for both genetic materials (Additional file 3) (*p *< 0.05). Consequently, recognition of RNA and DNA may suggest an involvement of TLR7 and TLR9, respectively, in *S. suis*-induced IL-1β production.

TLR activation triggers various intracellular signaling pathways of which the NF-kB pathway and MAPK p38, Jun N-terminal kinase (JNK) and extracellular-regulated kinase (ERK) are the most important [41]. To determine the role of these pathways in *S. suis*-induced IL-1β production, bmDCs were pre-treated with inhibitors (NF-κB inhibitor JSH-23, p38 inhibitor SB203580, MEK1/2 inhibitor U0126 or JNK inhibitor SP600125) or the vehicle (DMSO). Treatment with NF-κB inhibitor reduced secretion of IL-1β induced by both strains by approximately 50% (*p* < 0.01) (Figure 4D). Interestingly, different IL-1 production by strains P1/7 and SC84 was observed upon p38 inhibition: while p38 inhibitor had no effect on P1/7-induced IL-1β production, it significantly reduced SC84-induced IL-1β (*p *< 0.01) (Figure 4D). Finally, treatment with MEK1/2 inhibitor reduced IL-1β production by 90% for both strains (*p *< 0.01) while JNK inhibitor had no effect (Figure 4D). Consequently, except for p38 activation, *S. suis* strains P1/7 and SC84 use similar receptors and signaling pathways in the induction of IL-1β.

### *S. suis* induces inflammasome activation required for IL-1β maturation in a strain-dependent manner

**Figure 5:**
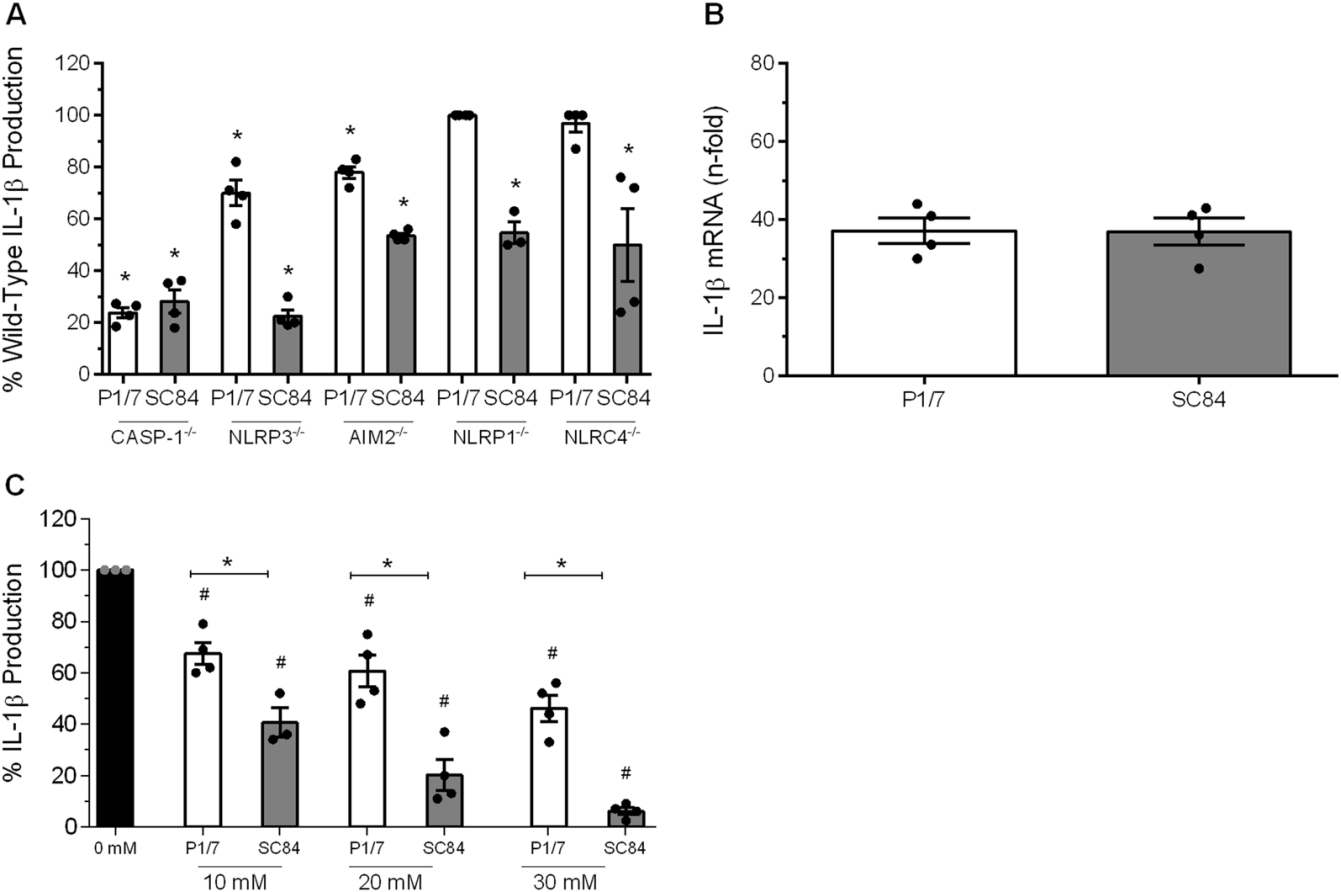
**Implication of caspase-1 and inflammasomes in** ***S. suis*-induced IL-1β bone marrow-derived dendritic cell (bmDC) production is strain-dependent.** **A** Percentage of IL-1β secretion by caspase-1 (CASP-1), NLRP3, AIM2, NLRP1 or NLRC4-deficient bmDCs induced by P1/7 (white bars) or SC84 (gray bars) after 16 h of incubation, in comparison to wild-type counterparts (normalized to 100%); **B** bmDCs were infected with P1/7 and SC84 strains for 6 h and IL-1β mRNA expression was measured by RT-qPCR. Data are presented as “fold” increase in mRNA expression relative to non-infected cells. **C** bmDCs were infected with either strain P1/7 or SC84 in the presence of different concentrations of KCl and IL-1β production was measured after 16 h by ELISA. Data are expressed as mean ± SEM (*n* = 4). *(*p *< 0.05) indicates a significantly difference with wild-type or non-treated bmDCs and ^#^(*p* < 0.05) indicates a difference between P1/7 and SC84.

To investigate whether maturation of *S. suis*-induced IL-1β requires caspase-1, bmDCs deficient for this enzyme was used. IL-1β production was reduced by more than 75% in caspase-1^−/−^ bmDCs (*p* < 0.01), indicating that it is essential for maturation of IL-1β following infection by both strains (Figure 5A). Subsequently, the role of different inflammasomes was investigated. Though several inflammasomes have been described, NLRP1, NLRP3, AIM2, and NLRC4 are the best characterized [19]. While NLRP3- or AIM2-deficiency resulted in a partial decrease of IL-1β release following stimulation with P1/7 (*p *< 0.05), there was no significant involvement of NLRP1 or NLRC4 (Figure 5A). Unexpectedly, IL-1β maturation induced by strain SC84 involved all four inflammasomes tested (*p *< 0.05), with a major implication of NLRP3 (Figure 5A). Specific activation was confirmed by measuring TNF, which is inflammasome-independent (Additional file 4).

Since inflammasome activation was different between the strains, we hypothesized that differential maturation, but not induction, of IL-1β could be responsible for the different levels induced in vitro. To confirm this hypothesis, IL-1β mRNA levels induced by both strains were evaluated. In accordance, levels of IL-1β expression induced by strains P1/7 and SC84 were similar (Figure 5B).

### *S. suis*-induced IL-1β production is blocked by additional extracellular potassium

Potassium (K^+^) efflux is a common denominator in the assembly and activation of inflammasomes [19, 42]. Consequently, production of IL-1β by *S. suis*-infected bmDCs exposed to increasing concentrations of extracellular K^+^, used to inhibit its efflux, was evaluated. Ten mM of extracellular K^+^ was sufficient to significantly inhibit IL-1β induced by both strains (*p* < 0.05) (Figure 5C). However, the effect was significantly greater for strain SC84 than for strain P1/7 (*p* < 0.01) (Figure 5C). Moreover, inhibition was dose-dependent, and this regardless of strain. Importantly though, at concentrations equal to or greater than 40 mM, the decreased levels of IL-1β observed were non-specific due to cell death (data not shown). Non-specific inhibition of IL-1β production by K^+^ efflux at concentrations between 10 mM and 30 mM was discarded by measuring TNF and IL-6 production, which were not inhibited (Additional file 5).

### Elevated suilysin production by the ST7 strain is required for efficient pro-IL-1β maturation by bone marrow-derived dendritic cells

**Figure 6:**
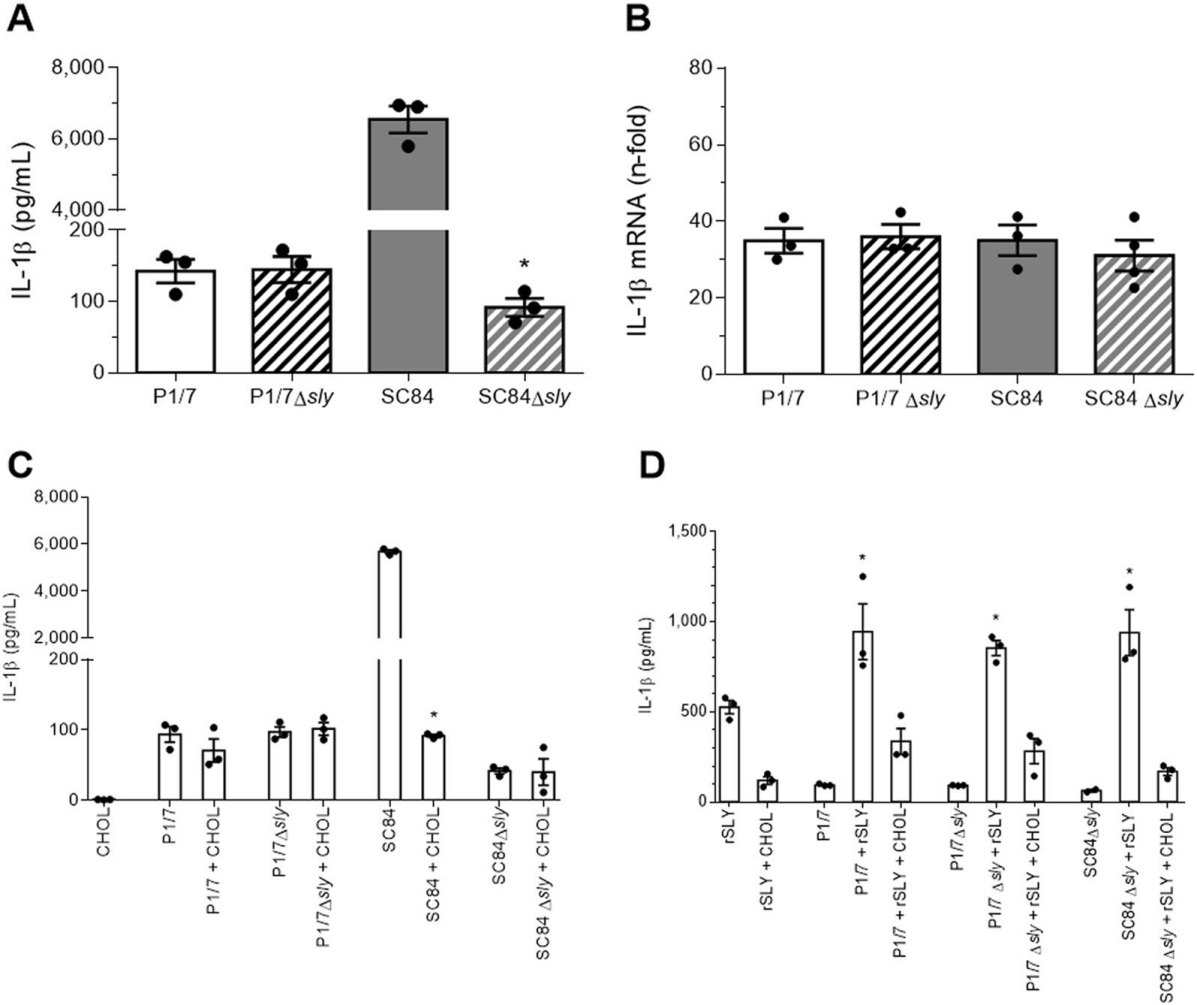
**Suilysin (SLY) is involved in the maturation of** ***S. suis*-induced IL-1β by bone marrow-derived dendritic cells (bmDCs). A** bmDCs were infected with the *S. suis* wild-type strains P1/7 and SC84 or their SLY-deficient mutants (∆*sly*) for 16 h and IL-1β release was measured by ELISA; **B** bmDCs were infected with the different wild-type and mutant strains for 6 h and IL-1β mRNA expression was measured by RT-qPCR. Data are presented as “fold” increase in mRNA expression relative to non-infected cells. **C** bmDCs were stimulated with the different strains of *S. suis* in the presence or absence of cholesterol (CHOL) for 16 h and IL-1β production was measured by ELISA; **D** bmDCs were infected with the different strains of *S. suis* alone, in combination with 5 μg/mL of rSLY or with 5 μg/mL of rSLY and CHOL. Data are expressed as mean ± SEM (n = 3). ^#^(*p* < 0.05) indicates a significant difference between SC84 and SC84Δ*sly* and *(*p* < 0.05) with bacteria alone.

Given that pore-forming toxins have the ability to induce K^+^ efflux [19] and that *S. suis* produces SLY, its role in IL-1β production was evaluated. ST7 strains were reported to have an increased SLY production in comparison to other serotype 2 strains [43], which we confirmed by measuring the hemolytic activity in P1/7 and SC84 supernatants (data not shown). To evaluate if SLY is implicated in *S. suis*-induced IL-1β release, *sly*-deficient isogenic mutants were constructed. No difference in IL-1β production was observed in the absence of SLY from strain P1/7 (Figure 6A). By contrast, absence of SLY from strain SC84 resulted in a significant decrease of secreted IL-1β (*p* < 0.001), suggesting that the higher levels of SLY produced by this strain are implicated in IL-1β production (Figure 6A). Moreover, IL-1β levels produced by SC84Δ*sly* were similar to those obtained with both wild-type P1/7 and P1/7Δ*sly*, suggesting that conserved components are responsible for the basal production of IL-1β by both strains (Figure 6A). In addition, the lack of differences in IL-1β mRNA expression between the wild-type and *sly*-deficient mutants confirms that SLY participates in pro-IL-1β maturation rather than in IL-1β induction (Figure 6B).

Since SLY is a cholesterol-dependent cytolysin, cholesterol inhibits its effects [30]. Consequently, cholesterol was added to bmDCs infected with the different *S.* *suis* wild-type and *sly*-deficient strains and did not itself induce IL-1β production (Figure 6C). While addition of cholesterol had no effect on IL-1β production by strains P1/7, P1/7Δ*sly* or SC84Δ*sly*, it significantly decreased production induced by the wild-type strain SC84 (*p *< 0.001), with residual levels being similar to those measured for P1/7, P1/7Δ*sly* and SC84Δ*sly* (Figure 6C).

Meanwhile, activation with rSLY alone induced moderate levels of IL-1β from bmDCs (Figure 6D). Since SLY has been suggested to induce TNF production following recognition by TLR4 [13], TLR4^−/−^ bmDCs were activated with rSLY. However, IL-1β production was TLR4-independent (Additional file 6). Meanwhile, addition of a non-toxic concentration of 5 μg/mL of rSLY resulted in a synergistic, but similar increase of IL-1β production by strains P1/7, P1/7Δ*sly* and SC84Δ*sly* (*p* < 0.01), which was abolished following treatment with cholesterol (Figure 6D).

### Strain-dependent role of IL-1 signaling in host survival during *S. suis* systemic infection

**Figure 7:**
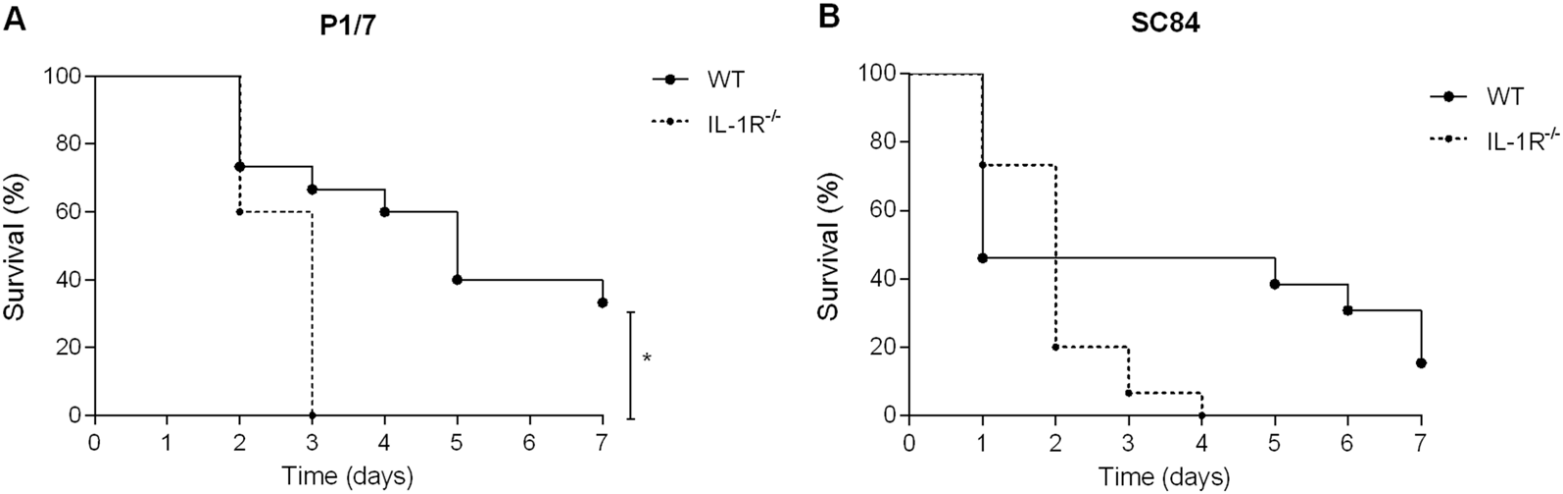
**Survival of wild-type (WT) and IL-1 receptor-deficient (IL-1R**^**−/−**^**) mice following** ***S.*** ***suis*** **systemic infection.** WT and IL-1R^−/−^ mice were inoculated with strain P1/7 (**A**) or SC84 (**B**) and survival was monitored. Data represent survival curves (*n* = 15). *(*p *< 0.05) indicates a significant difference between survival of WT and IL-1R^−/−^ mice.

Given the capacity of *S. suis* to induce IL-1 in vitro and in vivo, its implication in systemic inflammation and host survival was evaluated. Survival of IL-1R^−/−^ mice was significantly decreased in comparison to wild-type counterparts following infection with strain P1/7 (*p* < 0.01) (Figure 7A). Following infection with strain SC84, however, no statistical difference was observed between survival of wild-type and IL-1R^−/−^ mice (Figure 7B).

**Figure 8:**
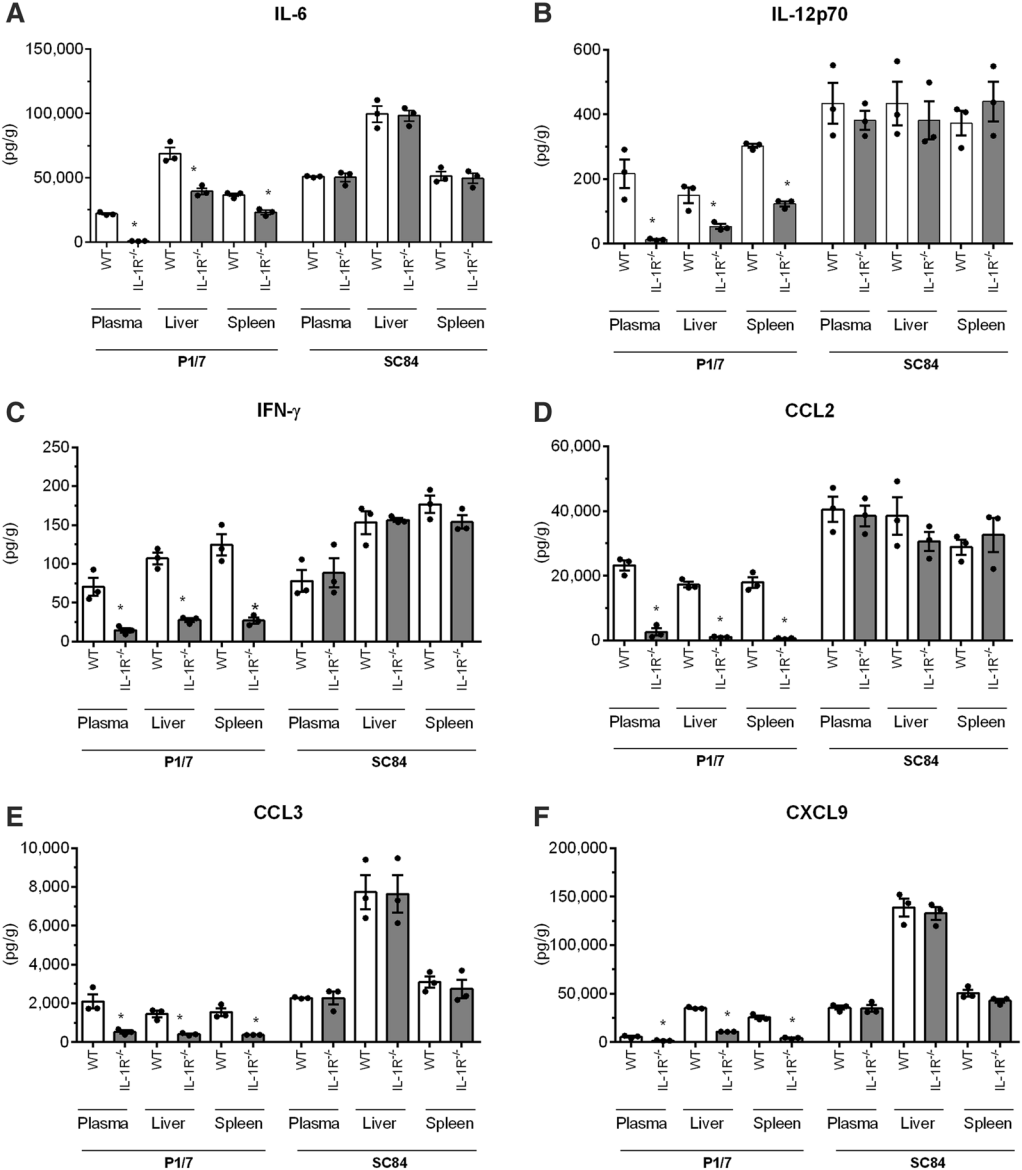
**Pro-inflammatory mediator production in plasma, spleen and liver during** ***S.*** ***suis*** **systemic infection.** Plasma, spleen and liver levels of IL-6 (**A**), IL-12p70 (**B**), IFN-γ (**C**), CCL2 (**D**), CCL3 (**E**), and CXCL9 (**F**) in wild-type (WT) and IL-1R^−/−^ mice 12 h following infection with strain P1/7 or SC84 strain. Data are expressed as mean ± SEM (*n* = 3). *(*p* < 0.05) indicates a significant difference between WT and IL-1R^−/−^ mice.

To better understand this difference, and since IL-1 is involved in initiation and amplification of inflammation, production of pro-inflammatory mediators in plasma, spleen and liver was evaluated 12 h pi. While IL-1α and IL-1β levels in WT and IL-1R^−/−^ mice were similar (Additional file 7), levels of IL-6, IL-12p70, IFN–γ, CCL2, CCL3, and CXCL9 were significantly lower in IL-1R^−/−^ mice compared with wild-type mice following infection with P1/7 (*p* < 0.05) (Figure 8). Following infection with strain SC84 however, levels of mediators were similarly exacerbated in both wild-type and IL-1R_–_^−/−^ mice (Figure 8).

**Figure 9:**
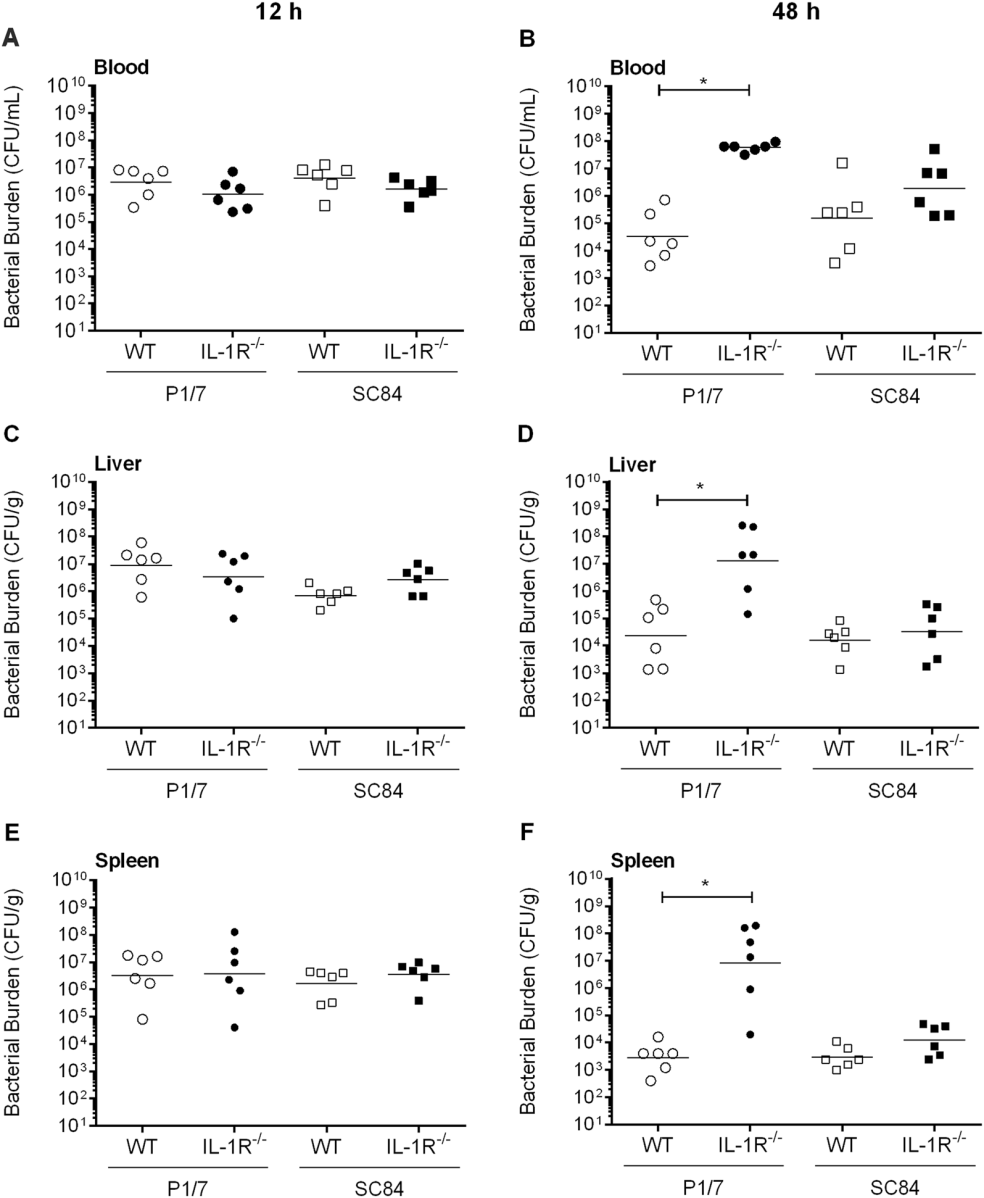
**IL-1 signaling is required for control of bacterial burden in blood, liver and spleen only for** ***S. suis*** **P1/7 strain.** Bacterial burden in blood (**A**, **B**), liver (**C**, **D**) and spleen (**E**, **F**) of wild-type (WT) and IL-1R^−/−^ mice infected with strain P1/7 or SC84 12 h (left panels) or 48 h (right panels) post-infection. A blood bacterial burden of 2 × 10^9^ CFU/mL, corresponding to the average burden upon euthanasia, was attributed to euthanized mice. Data represent the geometric mean (*n* = 6). *(*p *< 0.05) indicates a significant difference between WT and IL-1R^−/−^ mice.

Though inflammation is required for clearance of bacteria during *S. suis* systemic infection, it can also lead to host death if uncontrolled. Consequently, bacterial load in blood, spleen and liver was evaluated 12 h and 48 h following infection with strains P1/7 and SC84. No differences were observed between wild-type and IL-1R^−/−^ mice 12 h pi regardless of strain (Figure 9). Interestingly, 48 h p.i. following infection with P1/7 strain, bacterial burden was significantly higher in plasma, liver and spleen of IL-1R^−/−^ mice (*p *< 0.01) (Figure 9). By contrast, no differences were observed following infection with strain SC84, and this for all organs (Figure 9). Notably, bacterial load of wild-type mice infected with strain P1/7 or SC84 were similar at 12 h and 48 h pi.

## Discussion

Previous studies showed low levels of IL-1 in plasma after experimental infection with *S. suis*, in comparison with other important pro-inflammatory cytokines such as TNF or IL-6 [8, 35]. As such, the low levels of IL-1 observed herein in plasma after infection with both strains were not surprising and not related to the strain virulence level. Several factors could explain this near lack of IL-1, including its short half-life in plasma [44] and its association with other plasmatic proteins [45]. To our knowledge, however, there has been no other study evaluating plasma levels of IL-1 during infection by other streptococci using similar models systemic of infection. On the other hand, high levels of IL-1α and IL-1β were found in liver and spleen, which are two important filter organs. Previous studies with GBS also reported elevated levels of IL-1β in kidneys [23]. Therefore, although IL-1 cannot be found in plasma, activation of immune cells by infiltrating bacteria in liver and spleen might be responsible for the induction of IL-1, which remains locally.

To further analyze differences in IL-1 signaling between strains, in vitro studies with bmDCs and MΦs were performed. Interestingly, production by MΦ was somewhat delayed compared to that by bmDCs following infection with both strains. This seems to be a characteristic of streptococci, as it was also reported for GBS, GAS and *S.* *pneumoniae* [46–48], and might be due to a less efficient capacity of macrophages to process pro-IL-1β into its mature form. The high production of IL-1 in vivo, but only partial contribution of bmDCs and MΦ, suggests the implication of other immune cell types, of which neutrophils were also demonstrated to be a source during GBS infection [49].

Unlike with most other cytokines, IL-1β production is controlled by a two-step signaling process. Firstly, activation of PRRs such as TLRs leads to the transcription of pro-IL-1β. Subsequently, a second signal induces cleavage of the precursor into active IL-1β through capsase-1- and inflammasome-dependent maturation. The higher levels of IL-1β induced by strain SC84, in comparison to strain P1/7, suggested differential cell activation or processing mechanisms. However, the cellular activation leading to IL-1β production was similar for both strains, indicating that the recognized components are relatively well-conserved. In addition, production of IL-1β was MyD88-dependent and partially involved recognition of surface lipoproteins by TLR2. Comparable results were reported for GBS and *S.* *pneumoniae*, suggesting that recognized bacterial motifs might even be conserved amongst streptococci [46, 50]. Meanwhile, early studies suggested that certain toxins such as pneumolysin [51], listeriolysin O [52] and, more recently, SLY [13], may activate cells trough TLR4, an extracellular receptor which can signal via MyD88. However, the capacity of these toxins to activate TLR4 remains controversial. More recent studies with *S. pneumoniae* (including recombinant pneumolysin) showed that production of IL-1 was TLR4-independent [48, 53]. Indeed, we demonstrated that IL-1β production by bmDCs induced by SLY-positive P1/7 and SC84 strains as well as rSLY was TLR4-independent [11, 12]. Moreover, IL-1β production was TRIF-independent, confirming the lack of TLR4 implication, since this adaptor protein is engaged by the latter and TLR3. To our knowledge, this is the first study evaluating the role of TRIF during *S. suis* infection. Finally, although considered a classical extracellular pathogen, *S.* *suis* strains P1/7 and SC84 can be internalized, albeit at low rates, and the nucleic acids can be recognized by TLR7 and TLR9 [14]. In this study, we demonstrated that RNA and DNA have the capacity to induce IL-1β, and equally so for both strains. Importantly, production was only observed when DNA and RNA were complexed with DOTAP, suggesting that recognition occurs in a process similar to that of IFN-β, following internalization and degradation [14].

Following engagement of TLRs, activation of the MAPK and NF-κB signaling pathways results in the initiation of an inflammatory response leading to cytokine production. IL-1β production by bmDCs induced by both strains was dependent on the NF-κB and ERK pathways, but independent of JNK, similar to what has previously described for other cytokines induced by *S.* *suis* [54] and for other streptococci [55–57]. Meanwhile, IL-1β production induced by strain SC84, but not by strain P1/7, was also p38-dependent, suggesting differential mechanisms, possibly due to differences in bacterial components or virulence. Indeed, pore-forming toxin secretion and its induced osmotic stress were observed to modulate MAPK phosphorylation for listeriolysin O and streptolysin O [55, 58]. As such, the higher production of SLY by strain SC84 could be involved in p38 activation, which remains to be confirmed.

Receptors and pathways engaged by this pathogen could not explain the differences observed in IL-1β production between strains. Moreover, IL-1β gene induction confirmed that the first step involved in this production is similar. Consequently, the steps involved in its maturation were evaluated. Though IL-1β production induced by both strains depended on caspase-1, inflammasome activation was different between strains: while maturation of pro-IL-1β induced by strain P1/7 was only partially dependent on NLRP3 and AIM2, strain SC84 activated NLRP3 and, to a lesser extent AIM2, NLRP1 and, surprisingly, NLRC4. These differences in inflammasome activation between strains, both in the different inflammasomes activated and their implication levels, could explain the differential IL-1β levels produced by bmDCs. Although NLRP3 and AIM2 participate in IL-1β release by bmDCs and MΦ following infection by GBS and *S.* *pneumoniae* [46, 53], the implication of NLRP1 and NLRC4 have not yet been described following streptococcal infection [59]. The specific factors responsible for *S. suis*-dependent inflammasome activation are difficult to determine since all four inflammasomes can be activated by a wide range of molecules. Previous studies showed that NLRP1 could directly sense the protease activity of the *Bacillus* *anthracis* lethal toxin [60]. Although activation of NLRP1 by streptococcal pore-forming toxins has not yet been evaluated, elevated levels of the *S. suis* SLY might be involved in a similar process. Moreover, strain SC84, unlike strain P1/7, also possesses a type IV secretion system encoded by its 89 K pathogenicity island [61], which might be responsible for NLRC4 activation [62]. Regarding the AIM2 inflammasome, it has been previously shown to be activated by DNA [63]. In accordance, we observed that levels of IL-1β induced by DNA were higher than those induced by RNA when complexed with DOTAP. Future studies using porcine antigen presenting cells, namely DCs and MΦ, will be required to confirm that the mechanisms implicated in IL-1β induction and maturation are conserved between the two species, including the receptors and inflammasomes involved.

Previous studies showed that bacterial pore-forming toxins could play a role in inflammasome activation [42]. Herein, we showed that high levels of SLY production produced by strain SC84 play an important role and could explain the observed differences between strains. It has also been shown that SLY expression by ST7 strains (such as the SC84) is 6.3-fold greater at the mRNA level and 4.5-fold greater at the protein level than traditional ST1 strains, such as P1/7 [42]. However, no differences in the SLY produced by both type of strains are expected, since it was previously demonstrated that the *sly* gene is highly conserved between *S. suis* strains with a maximum diversity of 1.8% at the nucleotide level [64, 65], This further suggests that a minimal level of SLY (threshold) is required. In addition, the role observed for SLY is only associated with IL-1β maturation since levels of IL-1β mRNA where similar between strains. In other words, although cell activation by strains P1/7 and SC84 leads to similar levels of pro-IL-1β, the high levels of SLY produced by strain SC84 result in a more efficient maturation of this cytokine. Interestingly, though rSLY itself induced some IL-1β secretion from bmDCs, levels were similar to those observed when cells were stimulated with Alum alone. It has been previously suggested that Alum does not induce IL-1 synthesis but causes maturation and release of the IL-1β naturally synthetized by the cell [66]. The same principle could apply to rSLY.

Generation of a K^+^ efflux could be responsible for the mechanism by which SLY stimulates inflammasome activation, as previously described for pneumolysin and the β-hemolysin of GBS [23, 51]. Indeed, ion fluxes have been described to be involved in the assembly of the four inflammasomes evaluated, a fact that could explain SC84 pan-inflammasome activation [19, 42]. In our study, the addition of extracellular K^+^ inhibited *S. suis*-induced IL-1β production. Importantly, although only 10 mM of K^+^ was sufficient to reduce IL-1β production, the effect was greater for strain SC84. For strain P1/7, the K^+^ efflux generated could be due to other yet unknown bacterial mechanisms that are probably shared by other classical *S. suis* strains and responsible for “normal” inflammasome activation by this pathogen.

Following secretion, IL-1α and IL-1β bind their shared receptor, IL-1R, leading to cell activation, stimulation and secretion of diverse pro-inflammatory cytokines (positive feedback loop), recruitment of neutrophils and macrophages, and activation of killing mechanisms, amongst other effects [67]. Previous studies with GBS and *S. pneumoniae* showed a protective role of IL-1 during infection, with its absence altering bacterial clearance and survival [21–24]. In the case of *S. suis*, IL-1 signaling also plays a central and beneficial role following infection with the ST1 strain P1/7, which represents classical strains. Indeed, IL-1 signaling induced by this strain modulates the host innate immune response by increasing production of other pro-inflammatory mediators required for control of bacterial burden in blood and organs, which if unrestricted, causes host death. However, and similar to what has been described for type I IFN, the “protective effect” of IL-1 was not observed following infection with the highly virulent ST7 strain SC84 [14]. In fact, the levels of IL-1 induced by this strain were unable to modulate overall inflammation and host outcome since levels of inflammatory mediators were exacerbated despite similar bacterial loads than those in P1/7-infected mice. These results suggest that during *S. suis* systemic infection, the levels of induced inflammation play a critical role. In accordance, IL-1 signaling itself cannot counterbalance the exacerbated inflammation induced by SC84 strain, resulting in host death. Interestingly, this strain possesses additional virulence factors such as the 89 K pathogenicity island, which is responsible for a greater innate immune system activation, resulting in a cytokine storm [68]. It should be noted, however, that even in the presence of IL-1, mice still succumb to *S. suis* infection, though to a significantly lower degree and rate, demonstrating the need for a balanced and controlled inflammation. *S. suis*-induced IL-1 did not autoregulate itself, suggesting that levels induced in the first hours of infection are sufficient to activate the immune system. This is in accordance with results obtained during systemic infection with GBS, during which levels of IL-1β are similar between wild-type and IL-1R^−/−^ mice in kidneys, peritoneal lavage and brain [23].

**Figure 10:**
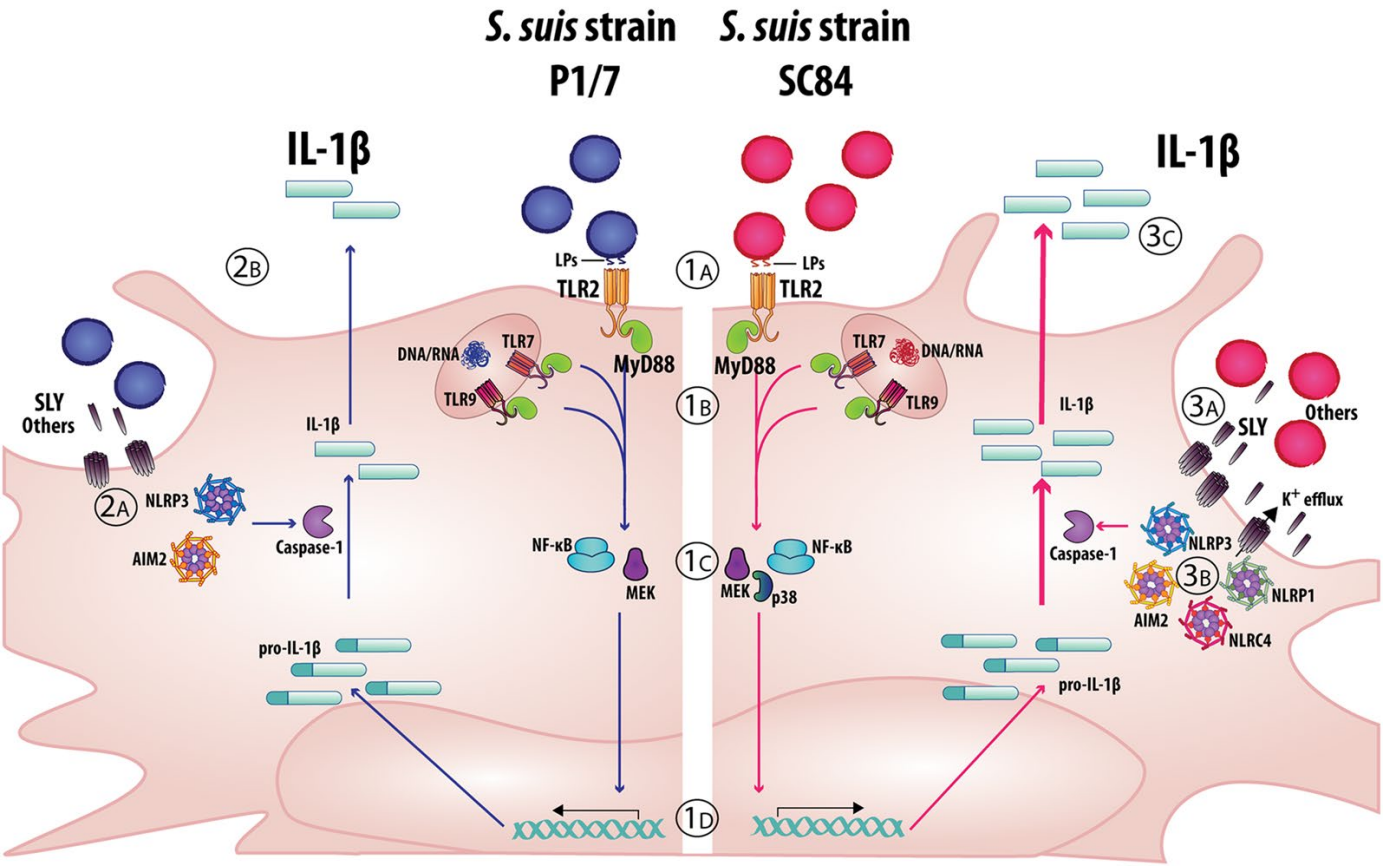
**Model of the mechanisms involved in** ***S. suis*-induced IL-1β production by bone marrow-derived dendritic cells (bmDCs).** 1A: Strain-independent recognition of *S. suis* by bmDCs requires MyD88-dependent signaling and partially involves TLR2 activation via recognition of surface lipoproteins (LPs); 1B: If internalized, *S. suis* DNA and RNA can induce the production of IL-1β, possibly via recognition by endosomal receptors TLR7 and TLR9; 1C: Recognition of *S. suis* leads to activation of the NF-κB and MEK pathways for both strains, alongside p38 for SC84; 1D: Strains P1/7 and SC84 induce comparable transcription of IL-1β mRNA; 2A: For strain P1/7, low levels of suilysin (SLY) and other not yet identified bacterial components lead to partial NLRP3 and AIM2 inflammasome activation; 2B: Caspase-1 cleavage leads to maturation of moderate levels of IL-1β that are then secreted; 3A and 3B: For strain SC84, secretion of high levels of SLY induces an important K^+^ efflux, that results in an activation of multiple inflammasomes, including NLRP3, NLRP1, AIM2, and NLRC4; however, other bacterial components could also influence this activation. 3C: Increased caspase-1 cleavage leads to a more efficient maturation of the pro-IL-1β, resulting in the secretion of high levels of IL-1β.

In conclusion, this study demonstrates that a classical (P1/7) and highly virulent (SC84) *S. suis* strain induce IL-1 in vivo, but only in internal organs. While both strains similarly activate innate immune cells due to conserved bacterial components such as LPs, high levels of the pore-forming toxin SLY play an important role in IL-1β maturation via activation of the NLRP1, NLRP3, AIM2, and NLRC4 inflammasomes. Based on these results, a model of the mechanisms involved in *S.* *suis*-induced IL-1β production by bmDCs is proposed (Figure 10). Globally, *S. suis*-induced IL-1 plays a beneficial role during systemic infection by initiating the inflammatory cascade. Beyond a certain threshold, however, *S. suis*-induced inflammation cannot be counterbalanced by this signaling, making it difficult to discriminate its role. Moreover, a better understanding of the underlying mechanisms involved in inflammation and subsequent bacterial burden will be necessarily to help develop control measures for this important porcine and zoonotic agent. Amongst these, future in vitro studies using porcine cells and in vivo studies in pigs will be necessary to confirm the results obtained herein using mice.

## Additional files

**Additional file 1.** **Oligonucleotide primers used in this study.**

**Additional file 2.** **IL-1α release from bone marrow-derived dendritic cells (bmDCs) and macrophages (MФ) stimulated with** ***S. suis*** **is strain-dependent.** IL-1α kinetics as measured by ELISA following infection of bmDCs (A and C) or MФ (B and D) with strain P1/7 (white bars) or SC84 (gray bars). Non-stimulated cells served as negative control (C-). Data are expressed as mean ± SEM (*n* = 4).

**Additional file 3.** **Addition of Alum enhances** ***S. suis*** **nucleic acid-induced IL-1β production by bone marrow-derived dendritic cells (bmDCs).** IL-1β production by bmDCs following activation with 1 µg of *S. suis* RNA or DNA from strains P1/7 and SC84 in the presence of Alum. Data are expressed as mean ± SEM (*n* = 3). *(*p* < 0.05) indicates a significant difference with negative control (elution buffer).

**Additional file 4.** ***S. suis*-induced TNF production by bone marrow-derived dendritic cells (bmDCs) is inflammasome-independent.** Percentage of TNF secretion by caspase-1 (CASP-1), NLRP3, AIM2, NLRP1 or NLRC4-deficient bmDCs induced by strain P1/7 (white bars) or SC84 (gray bars) after 16 h, in comparison to wild-type counterparts (normalized to 100%). Data are expressed as mean ± SEM (*n* = 3).

**Additional file 5.** ***S. suis*-induced IL-6 and TNF secretion by bone marrow-derived dendritic cells (bmDCs) is independent of additional extracellular potassium (K**^**+**^**) concentrations.** bmDCs were infected with either strain P1/7 or SC84 in the presence of different concentrations of KCl and IL-6 (**A**) or TNF (**B**) production was measured after 16 h by ELISA. Data are expressed as mean ± SEM (*n* = 3).

**Additional file 6.** **IL-1β production by recombinant suilysin (rSLY) is Toll-like receptor (TLR) 4-independent.** IL-1β secretion by wild-type and TLR4^−/−^ bone marrow-derived dendritic cells stimulated with rSLY (5 μg/mL) for 16 h. Data are expressed as mean ± SEM (n = 3).

**Additional file 7.** **IL-1 does not modulate its own production following** ***S. suis*** **infection.** Spleen and liver levels of IL-1α (A and B) and IL-1β (C and D) in wild-type (WT) and IL-1R^−/−^ mice 12 h following infection with strain P1/7 or SC84. Data are expressed as mean ± SEM (n = 5).

## Data Availability

The data and materials not presented in this manuscript are available from the corresponding author upon request.

## Abbreviations

AIM2: absent in melanoma 2
bmDC: bone marrow-derived DC
CFU: colony-forming unit
CCL: C–C motif chemokine ligand
CXCL: C–X–C motif chemokine ligand
DC: dendritic cell
DMSO: dimethyl sulfoxide
ELISA: enzyme-linked immunosorbent assay
ERK: extracellular-regulated kinase
GAS: Group A *Streptococcus*
GBS: Group B *Streptococcus*
IFN: interferon
IL: interleukin
IL-1R: interleukin-1 receptor
JNK: Jun N-terminal kinase
LB: Luria–Bertani
LP: lipoprotein
LTA: lipoteichoic acid
MФ: macrophage
MAPK: mitogen-activated protein kinase
MyD88: myeloid differentiation primary response 88
NF-ĸB: nuclear factor-kappa B
NLR: NOD-like receptor
NLRC4: NLR family CARD domain-containing protein 4
NLRP: NLR family pyrin domain-containing
NOD: nucleotide-binding oligomerization domain
PCR: polymerase chain reaction
PRR: pattern recognition receptors
rSLY: recombinant suilysin
SLY: suilysin
ST: sequence type
THA: THB agar
THB: Todd Hewitt broth
TLR: Toll-like receptor
TNF: tumor necrosis factor
TRIF: TIR-domain-containing adapter-inducing IFN-β
