# Supplementary material for: Interleukin-1 signaling induced by Streptococcus suis serotype 2 is strain-dependent and contributes to bacterial clearance and inflammation during systemic disease in a mouse model of infection
Source: Vet Res. 2019 Jul 1;50:52. doi: 10.1186/s13567-019-0670-y (PMC6604435; doi:10.1186/s13567-019-0670-y)
Supplement: Supplementary file 1 — Additional file 1. Oligonucleotide primers used in this study. [file 13567_2019_670_MOESM1_ESM.docx]

**Additional file 1. Oligonucleotide primers used in this study**

| Primer name | Sequence (5’ – 3’) |
| --- | --- |
| *sly* -ID1 | GAAGTGACTGCTGACAAGATGC |
| *sly* –ID2 | GCGCAATACTGATAAGCGTTGG |
| *sly* -ID3 | CAATCCAGGTGTTCCGATTTCG |
| *sly* -ID4 | TGCAGGAGATCTGCGACTAAG |
| *sly* -ID5 | CGCAGATATGCGGATGAAG |
| *sly* -ID6 | CACCTCATCCGCATATGCCAAACTGACTAT |
| *sly* -ID7 | ATAGTCAGTTTGGCATATGCGGATGAGGTG |
| *sly* –ID8 | CCAGTAAGAGACCAGCAACAGG |
| PT101 *sly* | F: GCGC**CATATG**CATATGGATTCCAAACAAGATATTAAT^1^  R: CGCG**GGATCC**TTACTCTATCACCTCATCCGC^2^ |
| *Atp5b* | F: ACC AGC CCA CCC TAG CCA CC  R: TGC AGG GGC AGG GTC AGT CA |
| *Gapdh* | F: CCC GTA GAC AAA ATG GTG AAG  R: GAC TGT GCC GTT GAA TTT G |
| *Il1a* | F: TCG GGA GGA GAC GAC TCT AA  R: TGA GTT TTG GTG TTT CTG GC |
| *Il1b* | F: AGG TCA AAG GTT TGG AAG CA  R: TGA AGC TAT GGC AAC TG |
| ^1^ NdeI site in bold  ^2^ BamHI site in bold |  |
