# Supplementary figures and images for: Interleukin-1 signaling induced by Streptococcus suis serotype 2 is strain-dependent and contributes to bacterial clearance and inflammation during systemic disease in a mouse model of infection
Source: Vet Res. 2019 Jul 1;50:52. doi: 10.1186/s13567-019-0670-y (PMC6604435; doi:10.1186/s13567-019-0670-y)

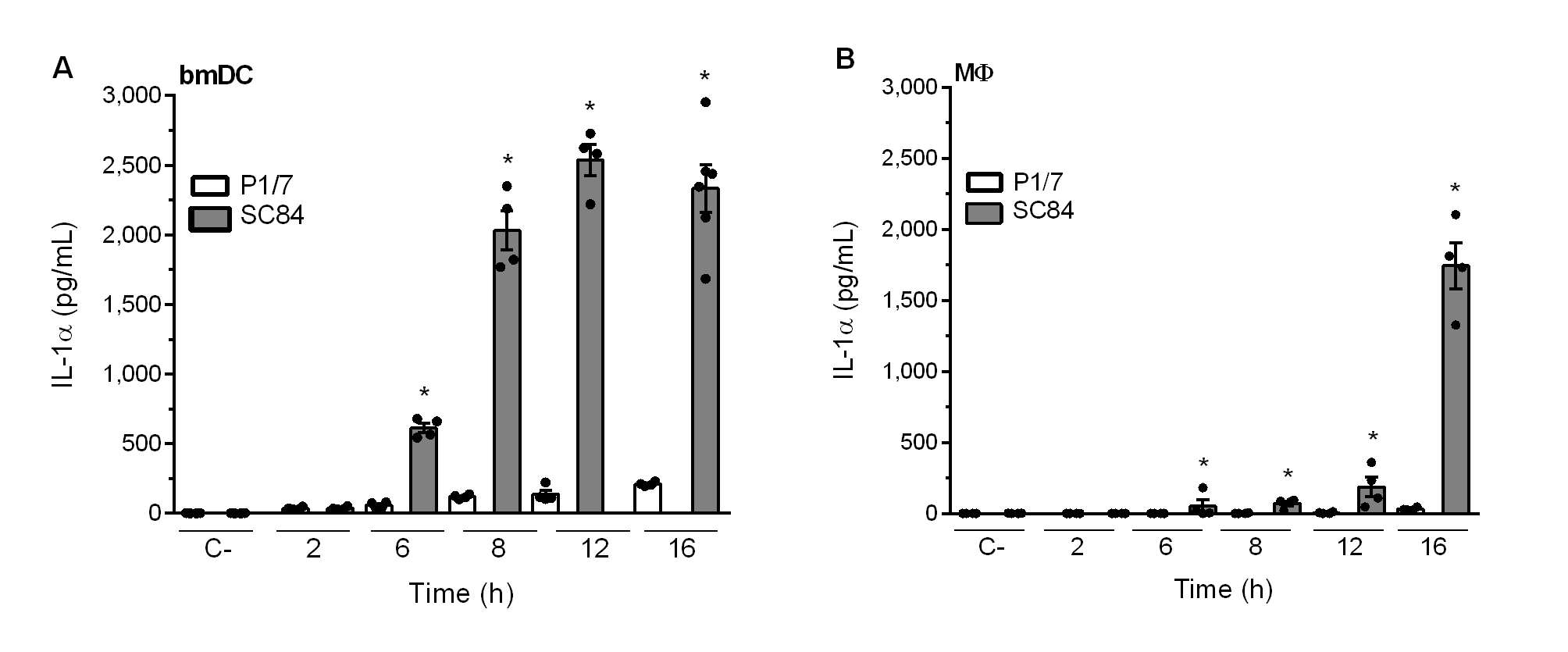

Supplement: Supplementary file 2 — Additional file 2. IL-1α release from bone marrow-derived dendritic cells (bmDCs) and macrophages (MФ) stimulated with S. suis is strain-dependent. IL-1α kinetics as measured by ELISA following infection of bmDCs (A and C) or MФ (B and D) with strain P1/7 (white bars) or SC84 (gray bars). Non-stimulated cells served as negative control (C-). Data are expressed as mean ± SEM (n = 4). [file 13567_2019_670_MOESM2_ESM.tif]

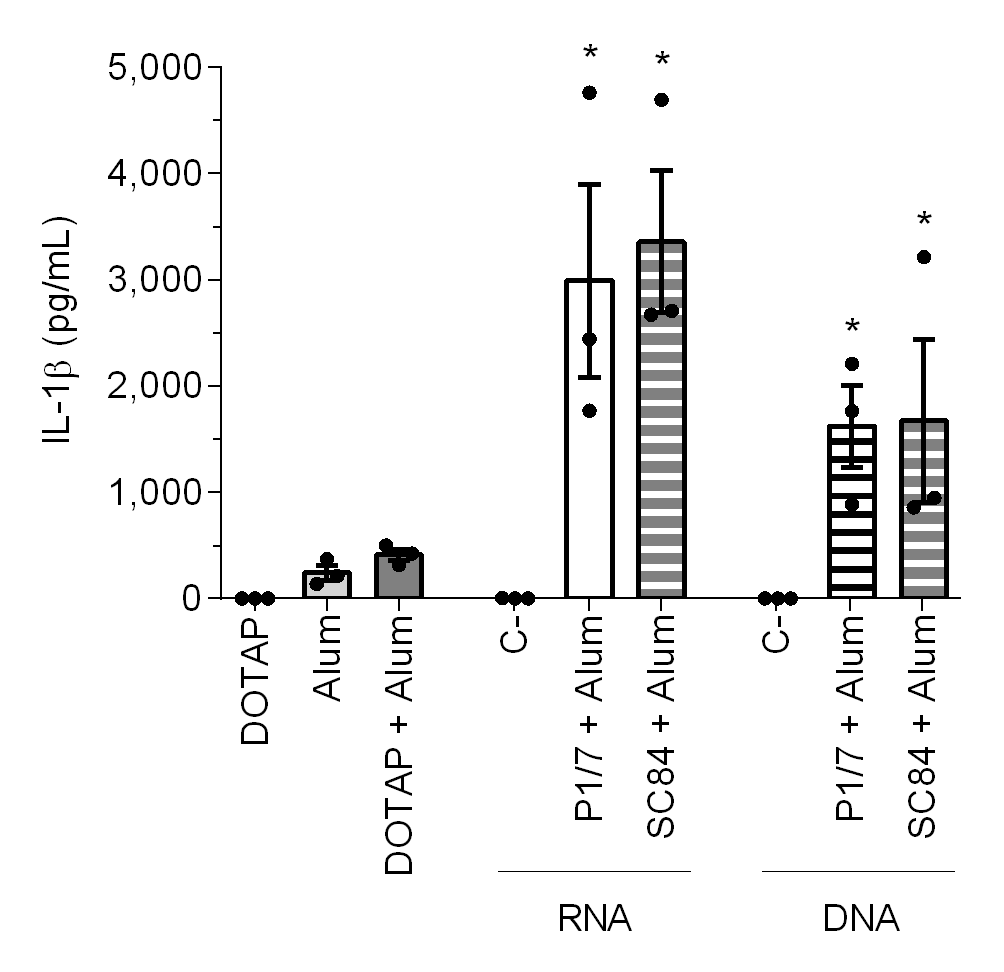

Supplement: Supplementary file 3 — Additional file 3. Addition of Alum enhances S. suis nucleic acid-induced IL-1β production by bone marrow-derived dendritic cells (bmDCs). IL-1β production by bmDCs following activation with 1 µg of S. suis RNA or DNA from strains P1/7 and SC84 in the presence of Alum. Data are expressed as mean ± SEM (n = 3). *(p < 0.05) indicates a significant difference with negative control (elution buffer). [file 13567_2019_670_MOESM3_ESM.tif]

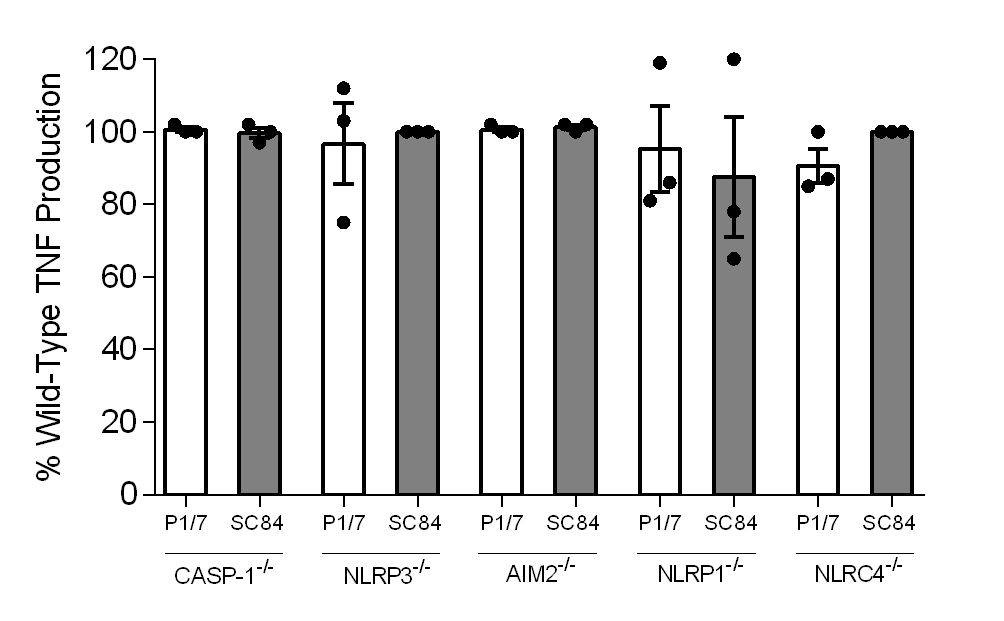

Supplement: Supplementary file 4 — Additional file 4. S. suis-induced TNF production by bone marrow-derived dendritic cells (bmDCs) is inflammasome-independent. Percentage of TNF secretion by caspase-1 (CASP-1), NLRP3, AIM2, NLRP1 or NLRC4-deficient bmDCs induced by strain P1/7 (white bars) or SC84 (gray bars) after 16 h, in comparison to wild-type counterparts (normalized to 100%). Data are expressed as mean ± SEM (n = 3). [file 13567_2019_670_MOESM4_ESM.tif]

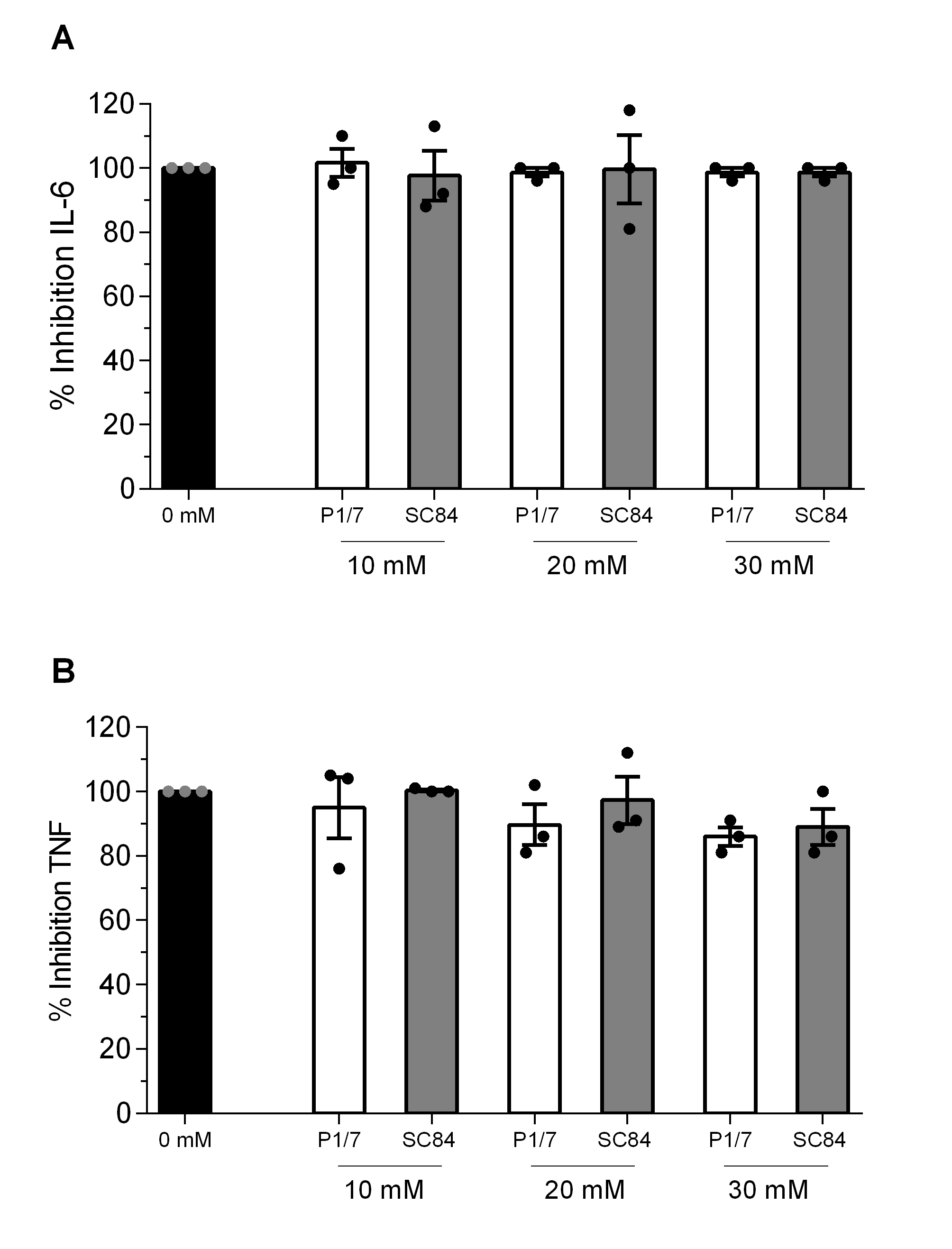

Supplement: Supplementary file 5 — Additional file 5. S. suis-induced IL-6 and TNF secretion by bone marrow-derived dendritic cells (bmDCs) is independent of additional extracellular potassium (K+) concentrations. bmDCs were infected with either strain P1/7 or SC84 in the presence of different concentrations of KCl and IL-6 (A) or TNF (B) production was measured after 16 h by ELISA. Data are expressed as mean ± SEM (n = 3). [file 13567_2019_670_MOESM5_ESM.tif]

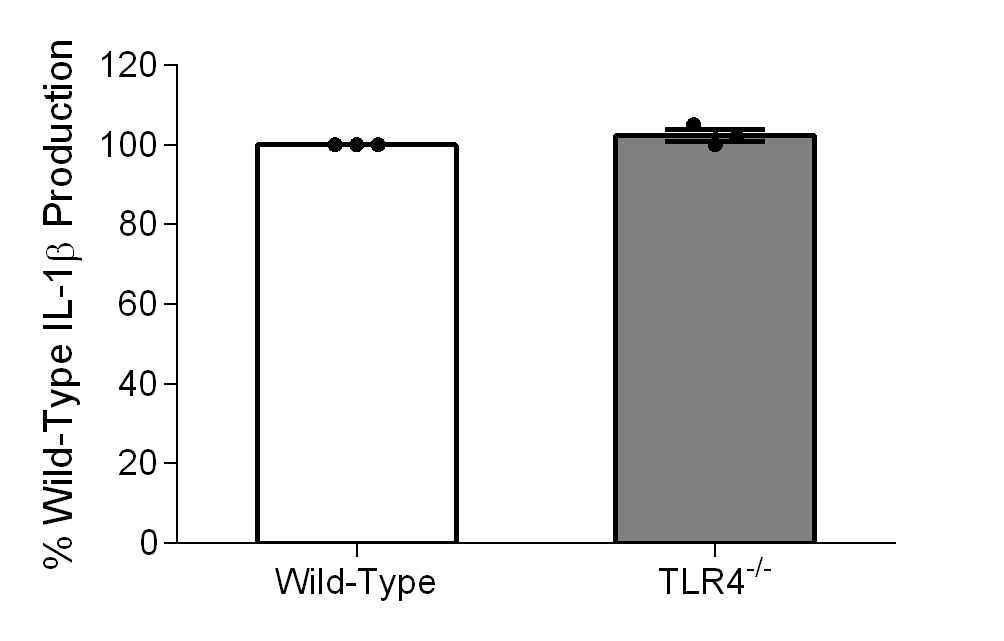

Supplement: Supplementary file 6 — Additional file 6. IL-1β production by recombinant suilysin (rSLY) is Toll-like receptor (TLR) 4-independent. IL-1β secretion by wild-type and TLR4−/− bone marrow-derived dendritic cells stimulated with rSLY (5 μg/mL) for 16 h. Data are expressed as mean ± SEM (n = 3). [file 13567_2019_670_MOESM6_ESM.tif]

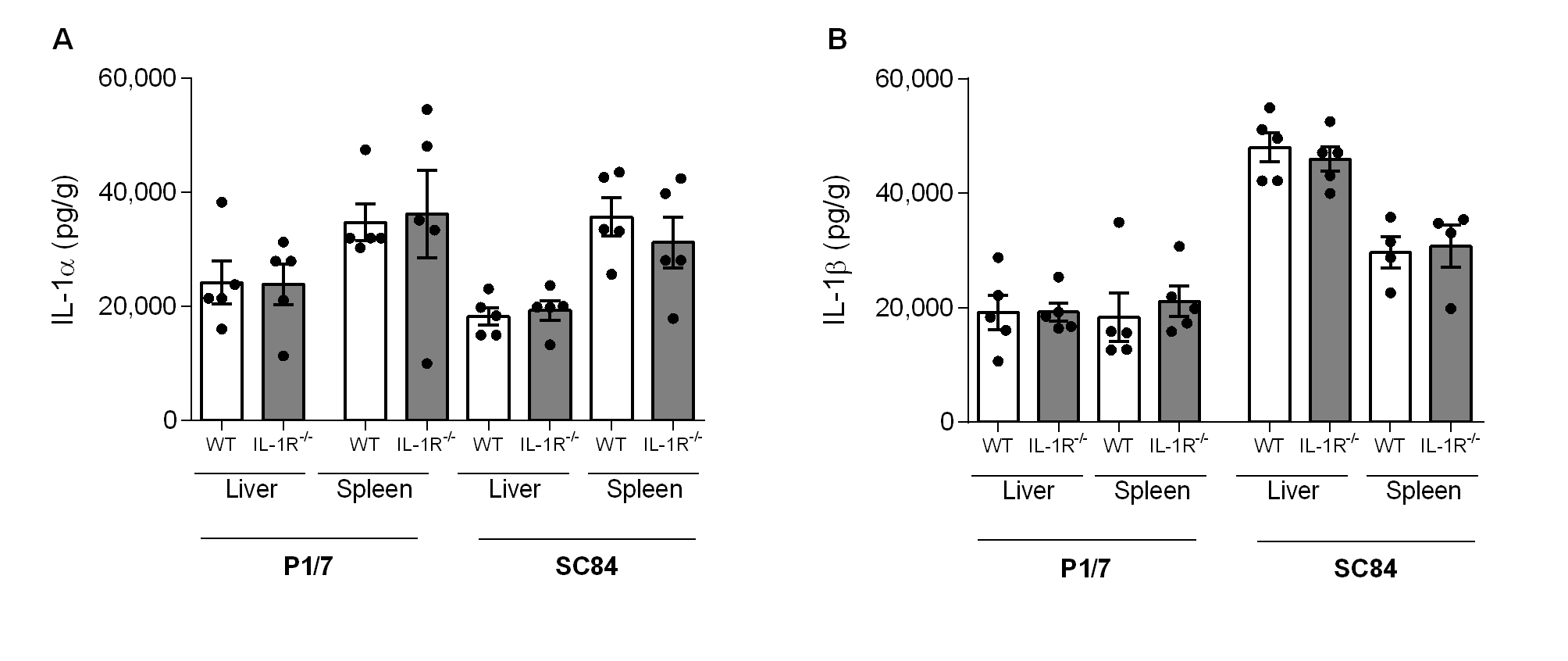

Supplement: Supplementary file 7 — Additional file 7. IL-1 does not modulate its own production following S. suis infection. Spleen and liver levels of IL-1α (A and B) and IL-1β (C and D) in wild-type (WT) and IL-1R−/− mice 12 h following infection with strain P1/7 or SC84. Data are expressed as mean ± SEM (n = 5). [file 13567_2019_670_MOESM7_ESM.tif]
